# A new leaf inhabiting ascomycete from the Jurassic (*ca* 170 Mya) of Yorkshire, UK, and insights into the appearance and diversification of filamentous *Ascomycota*

**DOI:** 10.1186/s43008-024-00162-9

**Published:** 2024-11-05

**Authors:** Ludovic Le Renard, Christine Strullu-Derrien, Mary Berbee, Mario Coiro

**Affiliations:** 1 Department of Botany, University of British Columbia, Vancouver, BC V6T 1Z4 Canada; 2 Science Group, The Natural History Museum, Cromwell Road, London, SW7 5BD UK; 3 Institut de Systématique, Évolution, Biodiversité (ISYEB), UMR7205, Muséum National d’Histoire Naturelle, CNRS, Sorbonne Université, 75005 Paris, France; 4 Department of Paleontology, University of Vienna, 1090 Vienna, Austria; 5 Ronin Institute for Independent Scholarship, Montclair, NJ 07043 USA

**Keywords:** Fossil fungi, *Pezizomycotina*, Cycadophytes, Environmental conditions, New taxon

## Abstract

Leaf-associated fungi, the fungi that depend on leaves to sporulate, have a rich Cenozoic record, however their earlier diversity is poorly characterized. Here we describe *Harristroma eboracense* gen. et sp. nov., a Middle Jurassic leaf-associated fungus colonizing the leaf cuticle of *Nilssonia tenuicaulis* (cycadophyte). To place our newly described species into a picture of the diversification of Mesozoic fungi, we reassess fossils with leaf-associated stromata in the context of fungal molecular phylogeny. Being melanized, with radiate stromata, and on leaves, *H. eboracense* and other fossils from the Jurassic and earlier periods are probably related to filamentous *Ascomycota* in the superclass *Leotiomyceta.* Characters needed for further resolution of leaf-associated fungal biology and classification, such as the presence of an ostiole for spore discharge and appressoria for entry into leaf tissue first appear in the Mesozoic fossil record. Among Early Cretaceous fossils, *Spataporthe taylorii* represents the oldest unambiguous evidence of perithecial *Sordariomycetes* while *Protographum luttrellii* and *Bleximothyrium ostiolatum* are the oldest *Dothideomycetes* thyriothecia. Environmental observations show that broad leaved gymnosperms (especially cycadophytes) growing in warm temperate wet forests might have been the first environment for the radiation of *Leotiomyceta.*

**Supplementary Information:**

The online version contains supplementary material available at 10.1186/s43008-024-00162-9.

## INTRODUCTION

*Ascomycota* is the largest and most diverse phylum within the *Fungi*. They play a crucial role in terrestrial ecosystems (e.g., symbiotic species forming mycorrhizas and nutrient cycling). They are of huge interest for medicine, industry, as commercial products, and they are cultivated as a source of food. In contrast, certain fungal species are notorious pathogens. Although the growing resolution of the *Ascomycota* tree of life brings new insights into their evolution, the timing of events leading up to their astonishing diversity is constrained by very few fossils.

The early fossil record of *Ascomycota* is poorly documented and controversial. Hyphae and spores that could potentially represent ascomycetes date back to the Silurian (Paleozoic Era) (Sherwood-Pike and Gray 1985). Lichenized fungi, a group mainly represented by ascomycetes, have been reported from the Precambrian and Palaeozoic, but these records are controversial (Lücking and Nelsen 2018). The earliest generally accepted fossil lichens are from the Early Devonian (Paleozoic Era) (Hawksworth 2015). Many fungi have been described from the Early Devonian Rhynie Chert [e.g., (Strullu-Derrien et al. 2014, 2016, 2018; Krings et al. 2016, 2018; Harper et al. 2017, 2020)]. Among these, *Mycokidstonia sphaerialoides* was described as an ascomycete ostiolate sporoma (Pons and Locquin 1981). Recently, Walker et al. (2021) redefined this fungus as a member of the *Glomeromycota*. While this reassignment seems dubious, there are not enough characters to attribute *M. sphaerialoides* to *Ascomycota*. *Paleopyrenomycites devonicus*, another fungus from the Rhynie Chert, has been observed protruding from the appendages (leaf-like enations) of the plant *Asteroxylon mackiei*. The fungal structures have been interpreted as perithecia and acervuli of thallic conidiophores (Taylor et al. 2005), although the morphological characters of this fossil are challenging to interpret. A plant pathogen, *Potteromyces asteroxylicola*, was recently discovered, also from this geological site*.* It has been attributed to *Ascomycota incertae sedis* (Strullu-Derrien et al. 2023) and reported to belong to an extinct lineage of ascomycetes. Strullu-Derrien et al (2023) reconsidered the sexual *P. devonicus*, its purported asexual morph (Taylor et al. 1999), and the dispersed hymenial layer of the Rhynie *Prototaxites taitii* (Honegger et al. 2018) and concluded that these can also be considered as *Ascomycota incertae sedis*. None of the fossils described as *Ascomycota* from the Rhynie Chert can be placed with confidence in the crown group, but together they provide the earliest compelling fossil evidence for stem group *Ascomycota* (Strullu-Derrien et al. 2023)*.* Dispersed spores and other evidence of a major *Ascomycota* radiation do not appear in the sedimentary record until much later in the Mesozoic (Berbee et al. 2020).

Many fungi in the extant *Ascomycota* are ‘leaf-associated’ (Hansford 1946; Hughes 1953; Marasinghe et al. 2023). Surface views of leaf-associated fossils were first reported from leaf compressions (Pampaloni 1902) before they were illustrated in greater detail from plant cuticle preparations (Edwards 1922; Rosendahl 1943; Cookson 1947; Rao 1958). They have also been found in long or cross sections of permineralized material (Suzuki 1910; Chrysler and Haenseler 1936; Van der Ham and Dortangs 2005). The views from sectioned material can be difficult to compare with surface views of fungi that result from cuticle preparations (Le Renard et al. 2021b).

One of the oldest putative leaf-associated fungi was photographed on leaf surfaces from the dispersed cuticle of a putative pteridosperm from the Lower Carboniferous (Paleozoic Era) (Hübers et al. 2011). Diverse, well-preserved leaf-associated fungi appear far later, on cuticles of plants from the Cenozoic Era (66 Mya onwards) (Dilcher 1965; Phipps 2007; Phipps and Rember 2004; Bannister et al. 2016; Conran et al. 2016). We hypothesize that the fungi of the Cenozoic represent products of extensive radiation of *Ascomycota* and predict that the earliest evidence of these radiations is to be found in the Mesozoic (252–66 Mya) record. Despite early reports and more recent papers by Sun et al. (2015) and Frolov (2018), few Jurassic (201–145 Mya) leaf-associated fungi have been described in the last 60 years, yet careful analysis of the rare leaf-inhabiting fossils from the Jurassic through the Cretaceous (Mesozoic Era) has some of the best potential for revealing early morphological diversification. By considering the characters of fossil fungi in the context of phylogenies of extant fungi, we can begin to infer when the clades of *Ascomycota* they represent had begun their radiation.

Here, we contribute to expanding knowledge of one of the rare, previously undescribed leaf-associated fungal fossils. We studied the cuticle of the cycadophyte *Nilssonia tenuicaulis* described by Harris (1964) from the Ravenscar Group (Middle Jurassic) of Yorkshire (UK). Harris (1961) observed fungi forming flat and appressed stromata on the cuticle of the fern *Phlebopteris polypodioides* from the Gristhorpe beds, and possibly also on the cuticle of the cycad *Ctenis reedi*, whose locality is uncertain but could also be from the Gristhorpe beds. In this paper, we describe *Harristroma eboracense* gen. et sp. nov., a fossil from the cuticle of *N. tenuicaulis*, a fungus superficially similar to one illustrated but not formally described by Harris (1961). We survey the literature and re-interpret the morphology of described fossil stromata. For phylogenetic placement of the fossils, we compare published descriptions of their characters with characters of lineages appearing in the genome-scale phylogeny of extant *Ascomycota* of Li et al. (2021). To infer the environmental conditions where leaf-associated fungi first developed, we discuss the paleoenvironment and composition of the Jurassic and Cretaceous floras in which they have been observed.

## METHODS

The specimen investigated was found on the cuticle of *N. tenuicaulis* mounted on slide n° NHMUK V25866a, housed in the collections of the Natural History Museum London since the work of Harris on the Yorkshire flora. The cuticle was macerated from the plant sample n° NHMUK V25866, from the Gristhorpe Plant Bed, an outcrop of the Gristhorpe Member in the Cloughton Formation (*ca* 170 Mya). We observed Harris' slide using a Nikon Eclipse LV100ND Microscope at the Natural History Museum London.

### Fungal analyses

We compare the fossil with fossil leaf-associated fungi forming circular to elliptical stromata as seen from above. Many such fossil stromata are superficial, on surfaces of leaves, and formed by radiating hyphae. Since stromata that form above leaf cuticle are uncommon in extant members of *Basidiomycota*, we hypothesize that the Mesozoic fossil record of stromata on leaves usually represent members of *Ascomycota*. We take a broad view, considering ‘stromata’ to include epicuticular hyphal aggregates or solid plates of mycelium as found in leaf-associated *Ascomycota* (Seifert et al. 2011; Ekanayaka et al. 2017; Maharachchikumbura et al. 2016; Hyde et al. 2013; Li et al. 2020; Wijayawardene et al. 2016). This broad view was necessary because in a fossil, radiate and appressed stromata in the same size range can represent somatic structures involved in the early stages of colonization (Upadhyay and Pavgi 1973), sexual thyriothecia, or asexual morphs of *Ascomycota* including pycnidia, aecia, and sporodochia (Sutton 1980; Nag Raj 1993; Ellis and Ellis 1997). We apply the somewhat narrower term ‘sporomata’ to structures with ostioles, pores or other indications of adaptation for dispersal of sexual or asexual spores.

For each fossil, we consulted the original illustration(s) and report characters that can be used for identification. Measurements of stroma and hyphal diameter are usually taken from Kalgutkar and Jansonius (2000) or its online version (Berbee et al. 2015), and missing dimensions for microstructures were measured from original publications in Fiji 2.1.0/1.53c (Schindelin et al. 2012). Characters noted for the fossils include whether the stromata are superficial, forming above the host cuticle, or immersed below the cuticle, or more deeply buried in the leaf. The initial development of the stromata could sometimes be inferred and recorded in superficial taxa by observing the relationship of superficial hyphae to stromata of increasing sizes (Le Renard et al. 2020a). Relationships of stromata to host tissues vary, so we recorded which stromata were associated with superficial hyphae or with the appressoria that are believed to contribute to penetrating into host cells. We considered whether sporomata had elongated slits (as found in thyriothecia, hysterothecia or lirellae) or round opening (ostioles) for spore release (as defined in Le Renard et al. 2020a). An ostiole is easy to recognize in a stroma when it is sharply delimited from surrounding cells that are smaller, darker, or differently shaped than other stromatal cells. In many of the fossils, openings interpreted as ‘ostioles’ were not sharply delimited and instead appeared to be central areas where fungal tissue was damaged and lost. If stromata are formed by hyphae that radiate from a central point of origin to the periphery and if the paths of the hyphae can be traced, the hyphal diameter may be seen to increase, decrease, or remain unchanged, serving as a character to distinguish species. Similarly, hyphal branching varies by species and may be isotomous (with roughly equal dichotomies) or anisotomous (producing new branches that are unequal in width). Hyphal septation is reported as ‘regular’ if adjacent hyphal segments appear similar in length, as ‘infrequent’ when scarcely present, or as ‘aseptate’ when absent. If hyphae are septate, hyphal cells may be long and nearly cylindrical, sinuous, or in straight, radial lines or short trapezoidal, rectangular, or square cells. In some fossils, lateral walls of hyphal cells bulge or are constricted asymmetrically and irregularly. Adjacent hyphae were recorded as ‘overlapping’ if they did cross over one another, and as ‘aligned’ if they did not.

**Fig. 1:**
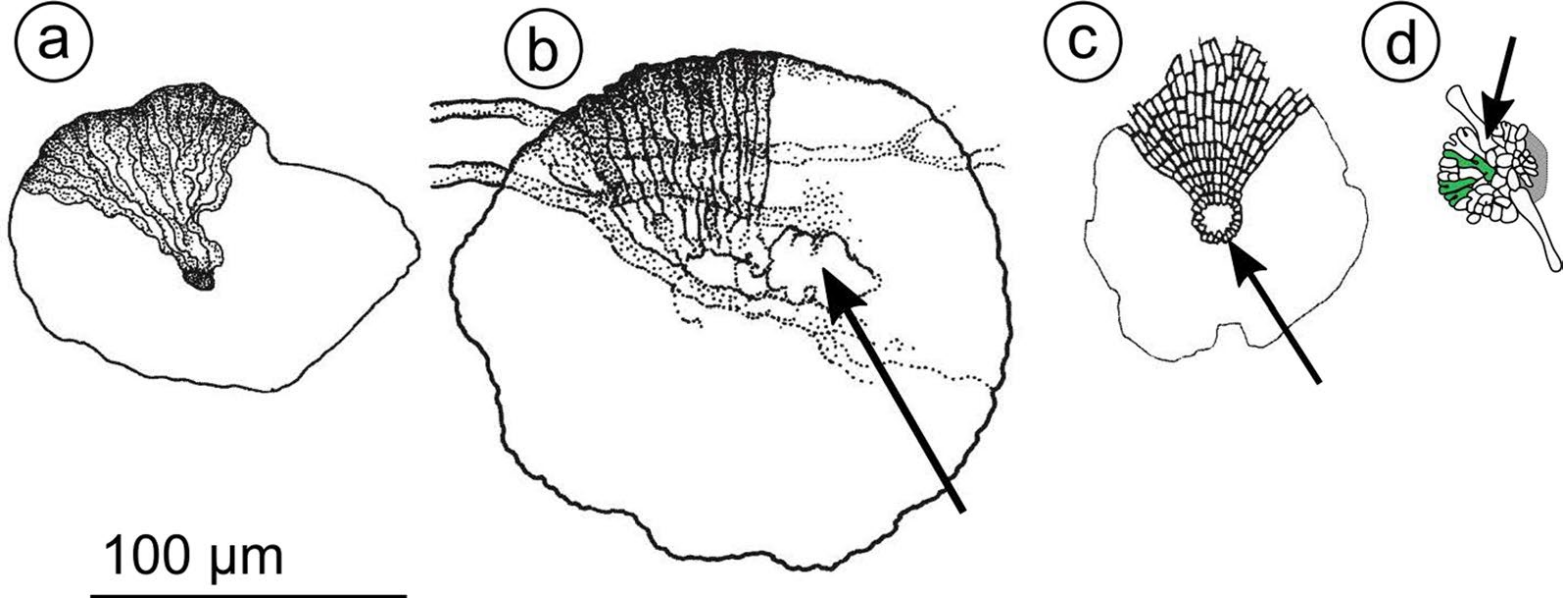
Four stromata from fossil fungi on leaf cuticle showing characters relevant to classification at different ranks; **a**–**b**, *Pezizomycotina, Leotimyceta* because of their radiate, melanized, and epicuticular stromata; **a** *Phragmothyrites doratophylli* (Early Cretaceous 121–100 Mya), stroma, no ostiole for spore release, cell walls of radiating hyphae bulge irregularly, hyphal diameter increases from centre to the stroma’s margin, transverse septa characteristic of genus cannot be resolved; **b** *Asterothyrites podocarpi* (Early Cretaceous 121–100 Mya) stroma, septa unresolved, with poorly differentiated opening (arrow), either tissue damage or an ostiole; **c** *Trichothyrites ostiolatus*, (Cenozoic, Oligocene–Miocene, 34–5 Mya) thyriothecioid stroma, *Dothideomycetes, Leotiomyceta* because of its radiate and ostiolate stroma, the ostiole (arrow) defined by a surrounding ring of small, isodiameteric cells; **d** *Asterina eocenica* (Cenozoic, Eocene, 49–37 Mya) thyriothecioid stroma, *Dothideomycetes, Leotiomyceta* because of its pattern of development indicated by a ‘generator hypha’ (arrow), which in extant taxa initiates the dichotomizing, radial stroma; hyphae in green highlight dichotomous branching. Image credits, drawings from Kalgutkar and Jansonius (2000) licensed under https://open.canada.ca/en/open-government-licence-canada: a, Pl. 21 Fig. 6; b, Pl. 22 Fig. 4; c, Pl. 23 Fig. 9; d redrawn from Dilcher Pl. 8 Fig. 64 (Dilcher 1965). Original photographs a-b, (Krassilov 1967); c (Cookson 1947) Fossil fungi from Tertiary deposits in the southern hemisphere

**Fig. 2:**
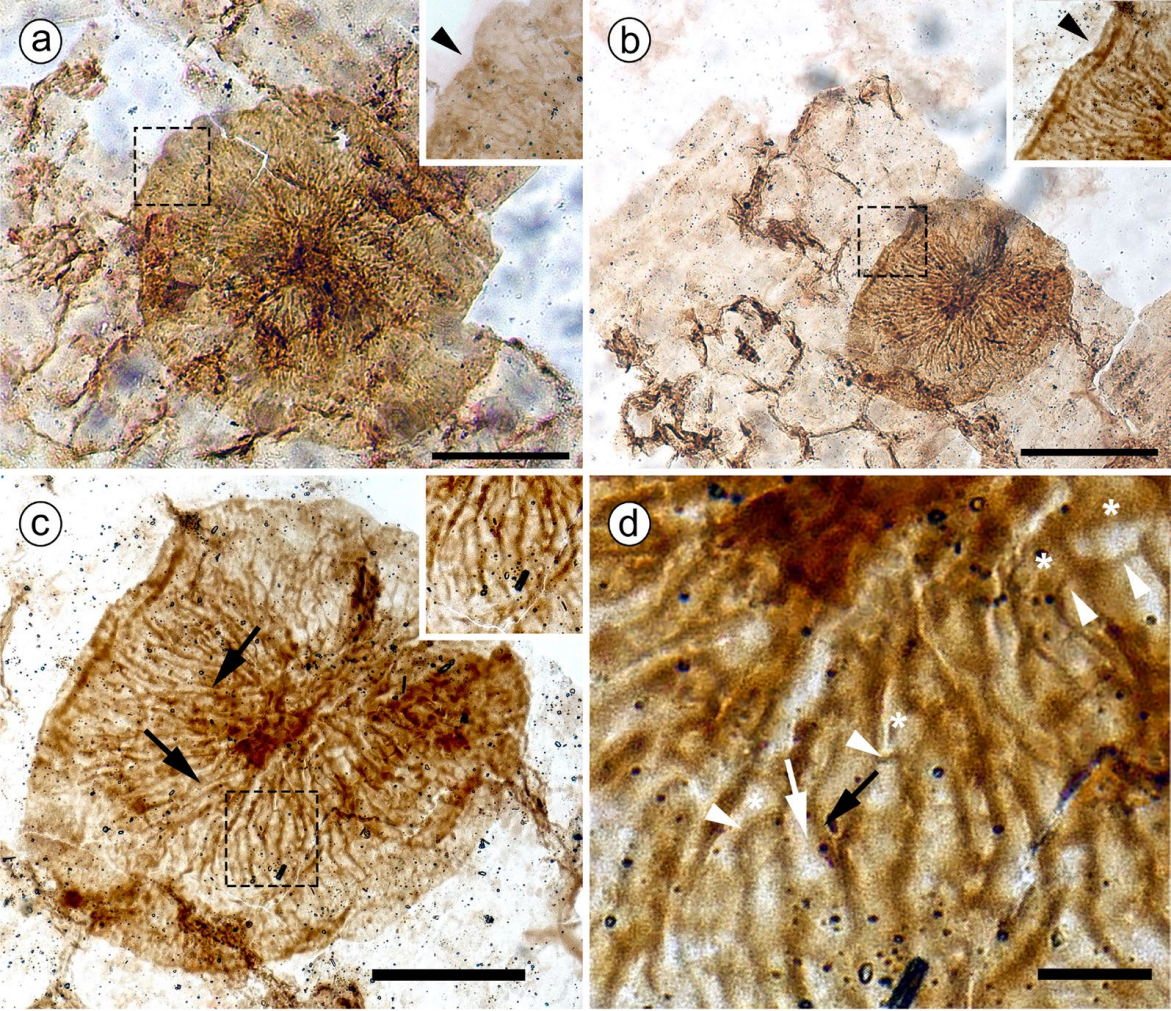
Holotype of *H. eboracense gen. et. sp. nov.* on cuticle fragments of *Nilssonia* slide n° NHMUK V25866a, insets correspond to dashed areas. a, b, Surface view of stromata, showing pinched margins (black arrowheads). **a** Circular stroma. **b** Elliptical stroma. **c** Elliptical stroma at higher magnification, with superficial overlapping hyphae (black arrows) and radially aligned hyphae (inset). **d** Elliptical stroma at higher magnification showing dichotomous branching (white asterisks) associated with septa (white arrowheads) and an example of hypha (black arrow) disappears below an adjacent one (white arrow). Scale bars, a, b = 100 µm, c = 50 µm and d = 10 µm

**Fig. 3:**
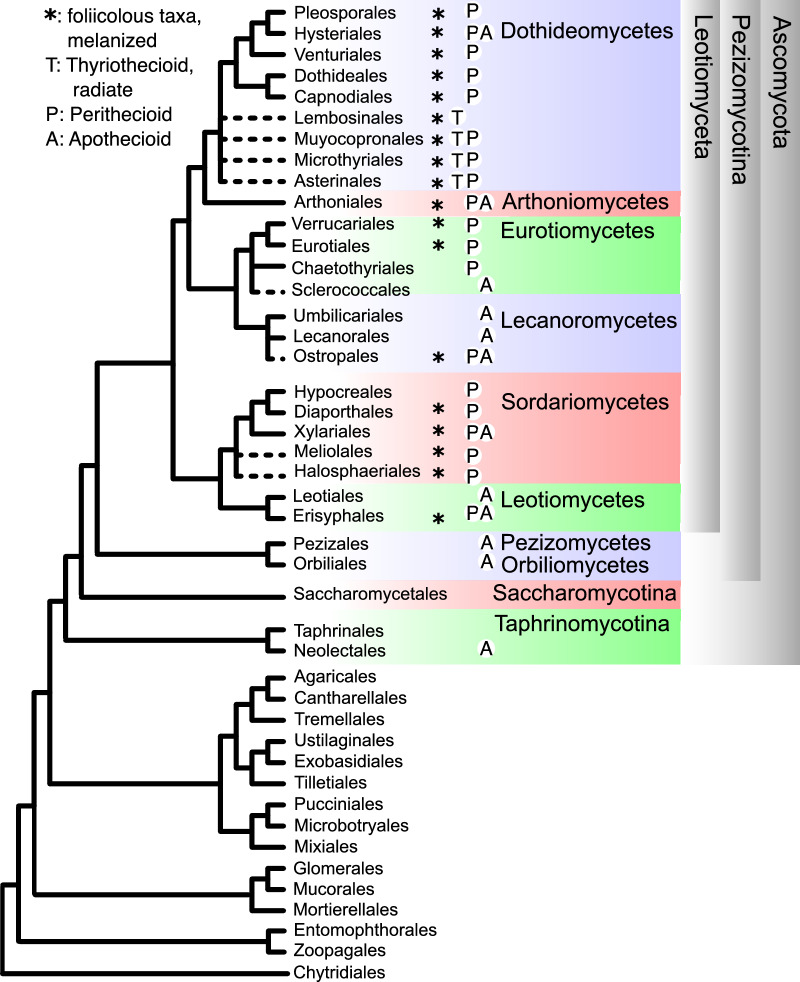
Summary of phylogeny from genome sequences (Li et al.) showing sporoma characters in extant orders of *Ascomycota*. Taxa on dashed branches were manually added to the topology based on their MycoBank (https://www.mycobank.org/, accessed 2022) classification

**Fig. 4:**
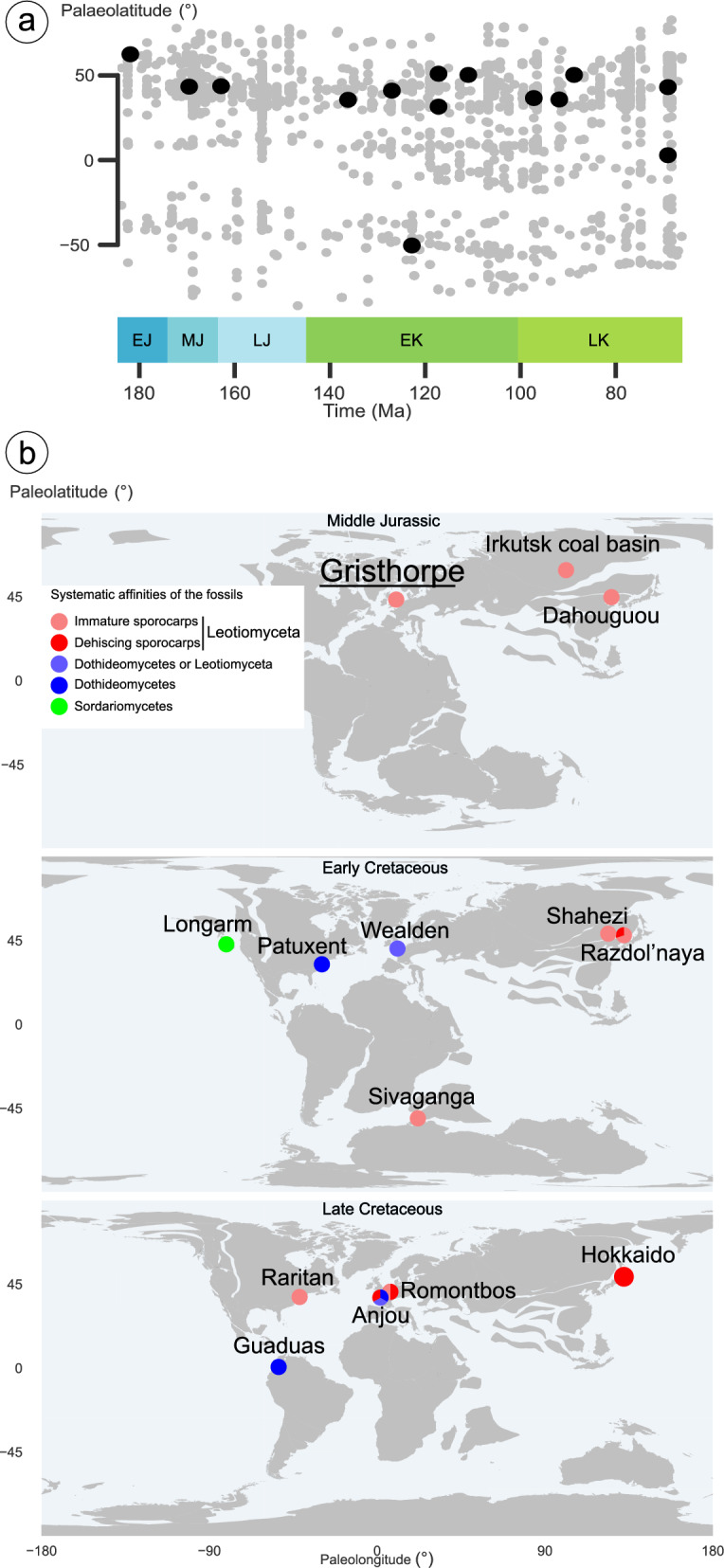
**a** Paleolatitude-through-time plot for the localities bearing leaf-associated fungi (black dots) and the plant bearing localities from the Paleobiology Database (grey dots) between the Jurassic and the Cretaceous. The graph shows the first appearance of leaf-associated fungi at the mid-high latitudes of the northern Hemisphere, and an expansion towards lower latitudes during the Late Cretaceous. EJ, Early Jurassic; MJ, Middle Jurassic; LJ, Late Jurassic; EK, Early Cretaceous; LK, Late Cretaceous. **b** Paleomaps showing the distribution of fossil leaf-associated fungi in the Middle Jurassic, Early Cretaceous, and Late Cretaceous. Pie charts represent the presence of different fungal structures and the proportion of each type in terms of proportional number of species

For comparison with fossils, our focus was on recognizing clades of extant taxa with melanized, leaf-associated species. For a broad taxon sampling, we surveyed published sporomata characters for at least one *Ascomycota* taxon from each order sampled by Li et al. (2021), and one from other orders that still lack genome sequence data (Additional file 1: Table S1). Fossils typically lack the asci or conidia that are key to morphological identification of living taxa. To match fossils to taxa, it is therefore necessary to draw on combinations of characters that may be found in both (Fig. 1a–d). Especially, shape of the stromata and the presence or absence of pre-formed openings that allow spore dispersal are important in relating fossil to living taxa (Fig. 1a–d). Thyriothecioid stromata, common on surfaces of extant and fossil leaves, include structures with a scutellum, that is a flat, shield-like dorsal layer of radially arranged (Fig. 1c, d) or tangled and interwoven hyphae as in *Mariusia andegavensis* (Pons and Boureau 1977: pl. I Figs. 1–5). Thyriothecioid fossils may represent asexual, conidium-producing pycnothyria or sporodochia. They may represent thyriothecia, ascus-producing sexual structures without a differentiated lower wall; or catathecia, similar to thyriothecia but with a differentiated lower wall. Evidence of differentiated cells surrounding an ostiole (Fig. 1c) or of preformed slits, or of regular pores for spore release would lead to interpretation of an extant or fossil scutellum as being thyriothecioid. Ostioles or other openings for spore release only appear in mature sporomata, and in their absence (Fig. 1d) other characters can sometimes contribute to interpreting a fossil. Evidence of early development of a scutellum from one or more ‘generator hyphae’, for example, supports identification of a fossil as being ‘thyriothecioid’ (Fig. 1d). Apothecioid sporomata include open cupulate sexual or asexual structures and true truffles. Perithecioid sporomata are broadly defined as globose or pyriform structures developing an ostiole at maturity; they may be sexual pseudothecia or perithecia, or asexual pycnidia. Cleistothecioid sporomata are any enclosed reproductive structures without a defined dehiscence mechanism. Some asexual structures without a dehiscence mechanism could not be classified following this scheme and are reported as ‘asexual sporomata’.

### Paleolatitude reconstruction

We estimated the paleolatitude of the fungi-bearing localities using the function ‘palaeorotate’ from the package palaeoverse (Jones et al. 2023) implemented in R version 4.2.1 (R Core Team 2018). We employed the PALEOMAP project model (Scotese and Wright 2018) for the rotations, and the ages of strata at the source localities were set as the midpoint of their Epoch borders. We then downloaded data from the Paleobiology Database selecting collections including plants (taxon ‘*Plantae’* from the Jurassic and the Cretaceous) as the background (http://paleobiodb.org/data1.2/colls/list.csv?datainfo&rowcount&base_name=Plantae&interval=Jurassic,Cretaceous&show=loc). Paleolatitude was calculated using the same rotation as employed for localities of fossil fungi, to allow direct comparison. The Paleobiology Database localities and the fungal localities were both plotted according to their age (using the midpoint between the maximum and minimum age) and their paleolatitude. We then used the functions ‘getmap’ and ‘mapast’ from the package mapast (Varela and Rothkugel 2018) to plot the localities on maps from the PALEOMAP project corresponding to the Visean (338 Mya), the midpoint of the Middle Jurassic (168.8 Mya), the midpoint of the Early Cretaceous (122.75 Mya), and the midpoint of the Late Cretaceous (83.25 Mya). The localities were then annotated with the proportion of *Ascomycota* lineages they contain, counted as the number of species associated with a particular group divided by the total amount of fungal species (summarized in Table 1).

**Table 1:** Melanized, leaf-inhabiting fossils sorted by age, then taxon name

Age, (stages, numerical age range)	Taxon (basionym); location; reference	Classification, taxonomic; stroma name	Host(s)	Associated mycelium	Stroma^1^ diam µm; dehiscence; location in host	Cell shape, arrangement in hyphae	Hyphal diam change, from stroma centre to edge?	Hyphae^2^ branching; diam (µm); Septa; Alignment
Lower Carboniferous (Middle Visean, ca 338 Mya)	Unnamed; Erzegebirge Basin Germany; (Hübers et al. 2011)—Figs. 1–2	Unknown; Possibly somatic	Pteridosperm	Possible subcuticular infection structure, no appressoria	100–170; na; Immrs	Fan shaped; in a repeated pattern, unicellular	Hyphal tips increase in width, branch, increase again	Iso- & Ani; Up to 9; Ab; Ol
Early Permian, (Barakar, 299–273 Mya)	‘Discoid stromata’; Deogarh Coalfield, Bihar India; (Bajpai and Maheshwari 1987)—Pl. 1, Fig. 1	*Leotiomyceta*; Circular stroma, radiate, type unknown	*Glossopteris* sp.	Traces?	36–248; na; supf?	Hyphal segments isosceles trapezoids, stroma unicellular?	hyphal tips increase in width, branch, increase again	Iso; ?;Ab, Al
Middle Jurassic (Pliensbachian to Aalenian, 192.9–170 Mya)	*Notothyrites* (?) sp. 1; Irkutsk Russia; (Frolov 2018)—Fig. 1a–d	*Leotiomyceta*; Thyriothecium?	*Czekanowskia baikalica* *Phoenicopsis irkutensis*, *Sphenobaiera angustiloba*, *Ginkgo* sp	None	60–200; RS?; Supf above host stomata	Sinuous, irreg asymm bulges & constrictions, radiating from centre to edge	**↓** Narrows at tips?	Dic?; na; na; na
Middle Jurassic (Pliensbachian to Aalenian, 190–170 Mya)	*Notothyrites* (?) sp. 2 Irkutsk Russia; (Frolov 2018)—Fig. 1e–f	*Leotiomyceta*; Circular stroma, type unknown	*Baiera majaea*, *Sphenobaiera* sp.	Scant	400–450; na; Supf	na	na	na; na; na; na
Middle Jurassic (Pliensbachian– Aalenian, 190–170 Mya)	*Notothyrites* (?) sp. 3 Irkutsk Russia; (Frolov 2018)—Fig. 1g–h	*Leotiomyceta*; Circular stroma, type unknown	*Baiera* sp.	None	400; na; Immrs	na	na	na; na; na; na
**Middle Jurassic (Lower Bajocian; ca 170 Mya)**	***Harristroma eboracense* gen. et. sp. nov.;** **Gristhorpe, UK;** **This study**	*Leotiomyceta*; Circular stroma, radiate, type unknown	***Nilssonia tenuicaulis***	None	175–270; na; Supf	**Sinuous irreg asymm bulges & constrictions, radiating from centre to edge**	**Unchanged**	Irreg; 2–5; Ir; Ol & Al
(Lower Bajocian; ca 170 Mya)	Unnamed; Gristhorpe, UK; (Harris 1961)—Fig. 1D–E	*Leotiomyceta*; Circular stroma, radiate? type unknown	*Phlebopteris polypodioides*	na	~ 250; na; Supf	sinuous?	na	na; na; na; Al
Middle–Upper Jurassic (Bajocian–Oxfordian, 170–157 Mya)	‘Microthyriaceous fungus’; Daohugou, China; (Sun et al. 2015)—Plates II and III	*Leotiomyceta*; Circular stroma, type unknown, central pseudo-parenchyma, periphery radiate	*Sphenobaiera* sp.	None	~ 300; na; Supf	Varies: central pseudo-parenchyma, sinuous hyphae radiating distally	Unchanged	Irreg & Pseudop; 1–4; Ir; Ol & Al
Early Cretaceous (mid-Valanginian, ~ 136 Mya ca.)	*Spataporthe taylori*; Longarm Formation, Canada; (Bronson et al. 2013)—Figs. 3–7	*Sordariomycetes*; perithecium or pycnidium	‘Conifer’	na, surface view not available	320–470; Os; Immrs	na	na	na; na; na; na
Early Cretaceous (Hauterivian–Aptian, 132–113 Mya)	‘Discoid stromata’; Sivaganga basin, India; (Bajpai and Maheshwari 1987)—Pl. 1, Fig. 3	*Leotiomyceta*; Circular stroma, radiate, type unknown	*Ctenozamites* sp	None	200–350; na; Supf	Isosceles trapezoids, radially aligned	**↑** Increase	Iso; 4–6; Re, Cn; Al
Early Cretaceous (Hauterivian–Barremian, 132–121.4 Mya)	*Stomiopeltites cretaceus*; Wealden Group, UK; (Alvin and Muir 1970)—Pl. I and II	*Dothideomycetes* or *Lecanoromycetes*; thyriothecium	*Frenelopsis* sp.	Supf mycelium	Up to 250; Os; Supf	Sinuous uniform diam, not radially aligned	Unchanged	Untr; 2–3.5; Re; Ol
Early Cretaceous (Aptian, 121.4–113 Mya)	*Bleximothyrium ostiolatum*; Patuxent Formation, USA; (Le Renard et al. 2021b)—Figs. 1–4	*Dothideomycetes*; radiate thyriothecium	Ginkgoales or Cycadales	Supf mycelium; appressoria; penetration pegs; subcuticular mycélium en palmettes	85–240; Os & ring of pseudoparenchyma; Supf	Sinuous, irreg lobes	↓ Decrease, tapers at tips	Iso & Pseudop; 1–4; Ir; Ol
Early Cretaceous (Aptian, 121.4–113 Mya)	*Microthyriacites fuxinensis*; Shahezi, China; (Zheng and Zhang 1986)—Pl. II, Figs. 8–9	*Leotiomyceta*; Circular stroma, radiate, type unknown	*Nilssonia sinensis* or *Ctenis lyrata*	none	50–130; na; Supf	Nearly cylindrical uniform, radially aligned	↑ Increase	Iso; na; Ab; Al
Early Cretaceous (Aptian, 121.4–113 Mya)	*Microthyriacites plicatus*; Shahezi, China; (Zheng and Zhang 1986)—Pl. II, Fig. 3–4	*Leotiomyceta*; Circular stroma, radiate, type unknown	*Nilssonia sinensis* or *Ctenis lyrata*	None	25–150; na; Supf	Very narrow isosceles trapezoids, nearly cylindrical, radially aligned	↑ Increase	Iso; na; Ir; ~ Cn; Al
Early Cretaceous (Aptian, 121.4–113 Mya)	*Notothyrites haizhouensis*; Shahezi, China; (Zheng and Zhang 1986)—Pl. II, Fig. 5	*Leotiomyceta*, *Dothideomycetes*?; Circular stroma, radiate, thyriothecium?	*Nilssonia sinensis* or *Ctenis lyrata*	None	50–60 × 70–110; Os?; Supf	Very narrow isosceles trapezoids, nearly cylindrical, radially aligned	Unchanged	ISO; na; na; Al
Early Cretaceous (Aptian, 121.4–113 Mya)	*Protographum luttrellii*; Patuxent Formation, USA; (Le Renard et al. 2020b)—Fig. 1	*Dothideomycetes*; radiate, immature thyriothecium	Pinales (Pinaceae or Podocarpaceae)	Supf mycelium, appressoria & penetration pegs	20–60; S?; Supf, from multiple generator hyphae	Sinuous, irreg; multi-lobed	Unchanged	Iso & pseudomonopodial; 1–3; Ir; Ol
Early Cretaceous (Aptian, 121.4–113 Mya)	*Stigmateacites simplex*; Shahezi, China; (Zheng and Zhang 1986)—Pl. II, Figs. 1–2	*Leotiomyceta*; circular stroma, type unknown	*Nilssonia sinensis* or *Ctenis lyrata*	None?	220–300; na; na	Sinuous, uniform diam, not radially aligned	Unchanged	na; na; na; na
Early Cretaceous (Aptian, 121.4–113 Mya)	*Stomatothyrium placocentrum*; Patuxent Formation, USA; (Le Renard et al. 2021a)—Figs. 1–3	*Dothideomycetes*; radiate thyriothecium	Pinales (Pinaceae or Podocarpaceae)	None	50–170; C; Supf, from columella from stomata	Sinuous, lobed; hyphae at same distance from centre ~ uniform diam	↓ Wider above central columella; uniform elsewhere	Iso & pseudomo; 1.5–4.5; Ir; Al & Ol
Early Cretaceous (Aptian–Albian, 121.4–100 Mya)	*Asterinites* sp.; Razdol’naya Basin, Russia; (Krassilov 1967)—Pl. II, Fig. 7	*Leotiomyceta*; Circular stroma, radiate, type unknown	*Athrotaxites sutschanicus*	None?	< 100; na; Supf	Radiate?	na	na; na; na; na
Early Cretaceous (Aptian–Albian, 121.4–100 Mya)	*Asterothyrites dictyozamiticola* (= *Notothyrites dictyozamiticola*); Razdol’naya Basin, Russia; (Krassilov 1967)—Pl. I, Figs. 1–2	*Leotiomyceta*; Circular stroma, radiate, type unknown	*Dictyozamites grossinervis*	None	Up to 90; na; Supf	Sinuous, slightly irreg with bulges & constrictions, radiating from central opening to edge	↑ Increase	ISO; na; na; Al
Early Cretaceous (Aptian–Albian, 121.4–100 Mya)	*Asterothyrites podocarpi* (= *Notothyrites podocarpi*); Razdol’naya Basin, Russia; (Krassilov 1967)—Pl. I, Fig. 3	*Leotiomyceta*; Circular stroma, radiate, type unknown	*Podocarpus harrisii*	None	183; na; Supf	Sinuous irreg asymm bulges & constrictions, radiating from central opening to edge	na	na; Up to 5; na; na
Early Cretaceous (Aptian–Albian, 121.4–100 Mya)	*Microthyriacites cephalotaxi* (= *Notothyrites cephalotaxi*); Razdol’naya Basin, Russia; (Krassilov 1967)—Pl. I, Figs. 4–7	*Leotiomyceta*; Circular stroma, radiate, type unknown	*Cephalotaxus ussuriensis*	None	30–120; na; Supf	Nearly rectangular to square isosceles trapezoids, radially aligned	↑ Increase	Iso; 4–6; Re, ~ Cn; Al
Early Cretaceous (Aptian–Albian, 121.4–100 Mya)	*Notothyrites* sp.; Razdol’naya Basin, Russia; (Krassilov 1967)—Pl. I, Fig. 9	*Leotiomyceta*; Circular stroma, radiate, type unknown	*Pterophyllum sutschanense*	na	~ 100; na; Supf	Radiate?	na	na; na; na; na
Early Cretaceous (Aptian–Albian, 121.4–100 Mya)	*Perisporiacites zamiophylli*; Razdol’naya Basin, Russia; (Krassilov 1967)—Pl. III, Figs. 3–5	*Leotiomyceta*; circular stroma, type unknown, opening by slits. Apothecium? Asexual?	*Zamiophyllum buchianum* & *Subzamites borealis*	na	320–550; slits; Partially Immrs?	na	na	Dense pseudop; na; na; na
Early Cretaceous (Aptian–Albian, 121.4–100 Mya)	*Phragmothyrites doratophylli* (= *Asterinites doratophylli*); Razdol’naya Basin, Russia; (Krassilov 1967)—Pl. II, Fig. 6	*Leotiomyceta*; Circular stroma, radiate, type unknown	*Doratophyllum sulcatum*	None	78–110; na; Supf	Sinuous, irreg asymm bulges & constrictions, radiating from centre to edge	Unchanged	na; 2–7; na; Ol & Al
Early Cretaceous (Aptian–Albian, 121.4–100 Mya)	*Phragmothyrites nilssonioptericola* (= *Asterinites nilssonioptericola*); Razdol’naya Basin, Russia; (Krassilov 1967)—Pl. II, Figs. 4–5	*Leotiomyceta*; Circular stroma, radiate, type unknown	*Nilssoniopteris rhitidorachis*	Thin traces	112–158; na; Supf	Narrow isosceles trapezoids, radiating from centre to edge	↑ Increase	Iso; Up to 6; Ir; Al
Early Cretaceous (Aptian–Albian, 121.4–100 Mya)	*Phragmothyrites otozamiticola* (= *Notothyrites otozamiticola*); Razdol’naya Basin, Russia; (Krassilov 1967)—Pl. I, Fig. 8	*Leotiomyceta*; Circular stroma, radiate, type unknown	*Otozamites* sp.	None	up to 50; na; Supf	Nearly rectangular to square isosceles trapezoids, radially aligned	↑ Increase	Iso; 4–5; Re, ~ Cn; Al
Early Cretaceous (Aptian–Albian, 121.4–100 Mya)	*Ussurithyrites araucariodendri*; Razdol’naya Basin, Russia; (Krassilov 1967)—Pl. I, Figs. 1–3	*Leotiomyceta*; thyriothecium, strap-shaped	*Araucariodendron heterophyllum*	None	95–112; L, poorly delineated opening; Supf	Nearly rectangular to square isosceles trapezoids, radially aligned in curving arc	Decrease? Wider in middle	Iso; ~ 2; Re; Al
Late Cretaceous (Cenomanian, 100–94 Mya)	*Mariusia andegavensis*; Anjou, France; (Pons and Boureau 1977)—Pl. I, Pl. II Figs. 1–7	*Dothideomycetes*; thyriothecium	*Frenelopsis* sp.	Supf mycelium, appressoria & penetration pegs Subcuticular mycélium en palmettes	45–100; Os; Supf	Sinuous, uniform diam, radiating but with much overlapping	Unchanged	Untr; 1–3.5; Re; Ol
Late Cretaceous (Cenomanian, 100–94 Mya)	‘champignon parasite des stomates’; Anjou, France; (Pons and Boureau 1977)—Pl. III, Figs. 5–8	*Leotiomyceta*; pycnidium	*Frenelopsis* sp.	None	10–50; Os?; Supf, from stomata	na	na	Pseudop; 1–3; Re; O
Late Cretaceous (Cenomanian, 100–94 Mya)	*Stomiopeltites cretaceus*; Anjou, France; (Pons and Bourreau, 1977)—Pl. II, Figs. 8–9; Pl. III, Figs. 1–4	*Dothideomycetes* or *Lecanoromycetes*; thyriothecium	*Frenelopsis* sp.	Scant supf mycelium, no appressoria	125–250; Os; Supf	Sinuous, uniform diam, not radially aligned	Unchanged?	Untr; 1–3; na; na
Late Cretaceous (Cenomanian, 100–94 Mya)	*Xylomites cycadeoideae*; Raritan, USA; (Chrysler and Haenseler 1936)—Figs. 1–7	Unknown; pseudoparenchymatous stroma	*Cycadeoidea peridermalis*	na, surface view not available	300 × 500; none; Immrs	na	na	Pseudop; Up to 3; na; na
Late Cretaceous (Turonian–Santonian 93.9–83.6)	*Pleosporites shirainus*; Hokkaido, Japan (Suzuki 1910)—Text-Figs. 2 and 3. Pl. VII, photo 6	*Leotiomyceta*; perithecium or pycnidium	*Cryptomeriopsi* *s mesozoica*	na, surface view not available	50–180; Os?; Immrs	na	na	Pseudop; 2–5; Re; na
Late Cretaceous (Maastrichtian, 76–66 Mya)	*Pteropus brachyphylli;* Romontbos quarry, Belgium; (Van der Ham and Dortangs 2005)—Pl. II, III and IV	*Leotiomyceta*; pseudoparenchymatous stroma	*Brachyphyllum patens*	None	~ 100; NA; Immrs, emerges from stoma	na	na	na; na; na; na
Late Cretaceous (Maastrichtian, 72–66 Mya)	*Callimothallus corralesense*; Guaduas, Colombia; (Doubinger and Pons 1975)—Fig. 1; Pl. I	*Dothideomycetes*; Circular, radiate sporodochia with dorsal pores	Eudicots	None	25–90; P; Supf, from spore	Nearly rectangular to square isosceles trapezoids, radially aligned	↑ Increase	Iso & pseudop; 2–5; Re; Al?
Late Cretaceous (Maastrichtian, 72–66 Mya)	‘Epicuticular stromata’; Romontbos quarry, Belgium; (Van der Ham and Dortangs 2005)—Pl. V	*Leotiomyceta*; thyriothecium	*Brachyphyllum patens*	na, surface view not available	~ 200; Os; Supf	na		na; na; na;

^1^**Stroma characters** as **diameter** in µm; **dehiscence** as *na*, not observed, or *C* circular slits, *L* lateral slits, *Os* ostiole, Os & ring of pseudoparenchyma, *P* pores; *RS* radial slits; *S* slits; **location in host** as *Immrs*, immersed under host cuticle, *Supf*, superficial above host cuticle. **Not available**, could not be resolved, *na*

^2^**Hyphae** as **Branching** *Irreg* irregular, *Iso* isotomous, *Ani* anisotomous, *Dic* dichotomous, *Pseudomo* Pseudomonopodial, *Pseudop* Pseudoparenchymatous, *Untr* untraceable;  **Hyphal diameter change** may be seen to ↑increase, ↓decrease, or remain unchanged; **Septation** as *Ab* absent, *Ir* irregular, *Re*, regular, *Cn* septa of tangential walls aligned concentrically, at least in part; **Hyphal alignment**, *Al*, aligned hyphae radiating, their sides touching without overlap, *Ol*, overlap, hyphae overlapping one another. **Not available**, could not be resolved, *na*

## RESULTS

We report fungal colonization on a leaf of *N. tenuicaulis*, a cycadophyte from the Middle Jurassic (*ca* 170 Mya) growing in a warm and wet temperate environment. The fungus is represented by stromata of different sizes but sharing similar morphology. Two stromata were observed on two fragments of the same plant cuticle (Fig. 2). They are circular to elliptical and show hyphae originating from a darkly pigmented central zone, and form an entire margin at the edge (Fig. 2, black arrowheads). Most hyphae show irregular dichotomous branching (Fig. 2, asterisks), septation (white arrowheads), and overlapping hyphae (black and white arrows). We compared our fossil to the fossil leaf-associated fungi already described and have not found any report of a similar fungus from deposits of this age. Compared to fossils of younger ages, the shape, branching pattern, and septation of the radiating hyphae that form its stromata distinguish our fossil from most others. In spite of their local irregularities, the average widths of the radial hyphae of our fossil remain approximately unchanged from their central point of origin to the edge of the stroma, and irregular branching rather than increasing hyphal width results in expanding of the circumference of the growing stromata. In other fossils, such as *Callimothallus corralesense* (Doubinger and Pons 1975), increase in stroma circumference resulted from an increase in hyphal diameter. Stromata of *Phragmothyrites doratophylli* (Fig. 1a), *Asterothyrites podocarpi* and *A. dictozamiticola* (Fig. 1b) (Krassilov 1967), as in our fossil, consist of radiating hyphae, which have sinuous cell walls with irregular bulges and constrictions (Table 1). They differ, however, in that they show no septation, and their branching patterns cannot be resolved from the original publication. We therefore describe our fossil as new (Table 1). We then report on the environment of growth shared by the fossil plants bearing epiphyllous fungi through the Palaeozoic to the Cenozoic, allowing us to predict the type of environment that might have given rise to the radiation of these fungi.

## TAXONOMY

Kingdom:* Fungi.*

Phylum: *Ascomycota.*

Class: *incertae sedis.*

Genus: *Harristroma* Le Renard, Strullu-Derrien, Berbee et M. Coiro, gen. nov.

MycoBank MB855085.

*Etymology:* The generic name honours the British palaeobotanist Thomas Maxwell Harris (1903–1983) who prepared the slide, and *-stroma* refers to the type of fungal structure that the fossil represents.

*Diagnosis.* The new genus is characterized by flat stromata made of sinuous hyphae radiating from a common darker central area through infrequent dichotomies. The prosoplectenchyma of overlapping, irregularly bulging hyphae where the average hyphal diameter changes little from the centre to the edge distinguishes this from other fossil taxa. It differs from the specimens of *Asterothyrites Microthyriacites* and *Phragmothyrites* (those that are well enough preserved to resolve septation) by its elongate cells and by the absence of isodiametric cells and/or pseudoparenchyma.

*Type species*: *Harristroma eboracense* Le Renard et al. 2024.

*Species*: *Harristroma eboracense* Le Renard, Strullu-Derrien, Berbee et M. Coiro, sp. nov.

(Fig. 2).

MycoBank MB855086.

*Etymology*: The specific epithet refers to *Eboracum*, the Roman name of the City of York, the historical main centre of the North Yorkshire region.

*Diagnosis.* Stromata are 170–270 µm in diameter. Hyphal widths irregularly and repeatedly bulge and narrow within a range of 2–6 µm in diameter as they branch or extend from stroma centre to its margin. Host is a cycadophyte.

*Type*::United Kingdom: Yorkshire: Gristhorpe, fossil in the Gristhorpe Member, Cloughton Formation (*ca* 170 Mya), two fossil stromata on a cuticle fragment of *N. tenuicaulis,* prep. *T.M. Harris* (BM [NHMUK] slide V25866a –holotype).

*Description*: *Stromata* on two fragments of the same cuticle differ in size but share the same micromorphology; the larger circular with an irregular outline, 270 µm diameter (Fig. 2a); the smaller slightly elliptical, 175 × 140 µm (Fig. 2b–d). *Hyphae* originating from a central zone of darkly pigmented material 20–45 µm across; irregularly septate; hyphal cells visible at the surface of the stromata 5–25 µm long with irregular dichotomous branching; width of sister branches 2–6 µm (Fig. 2d); the dorsal most superficial hyphae traceable from the central zone to the edge, overgrowing other hyphae (Fig. 2b); overlapping best seen where one hypha straddles over an adjacent one that disappears under the surface of the stroma (Fig. 2d); hyphal walls appear aligned with one another (Fig. 2c), or intertwined and overlapping with one other (Fig. 2d); hyphal tips align forming an entire margin, or a locally pinched margin where bulging, fan-shaped lobes of branching hyphae intersect (Fig. 2a, b).

*Notes:* Table 1 compares *H. eboracense* with other early leaf-associated stroma-forming taxa by considering fossil age, collection locality, putative classification, host(s), and characters of stromata and associated hyphae, based on published illustrations and descriptions.

Of the 36 fossils in Table 1, stromata of 22, including *H. eboracense,* were formed by radiating hyphae that originated in the centre of the stromata and that extended, with greater or lesser regularity, to the periphery. These contrast with stromata of the non-radiate species, which were variously pseudoparenchymatous as in *Xylomites cycadeoideae* (Chrysler and Haenseler 1936) or composed of slender, tangled hyphae as in *Stomiopeltites cretaceus* (Alvin and Muir 1970; Pons and Boureau 1977).

The organization of stromata in  radiate species further distinguishes morphological types. In *H. eboracense*, hyphae in the stromata are sinuous and septa irregular. In other species, such as *Microthyriacites cephalotaxi*, each radiating hypha was nearly straight, and regularly spaced septa delimited cells that were roughly isosceles tetrahedral, rectangular, or square. Of the fossils, 20, including *H. eboracense,* lacked a distinct ostiole (Fig. 2a, b) or had slit-like openings for spore release, reducing the number of characters available for comparison and limiting the precision of their classification. In the Jurassic and many of the Cretaceous fossils, absence of fungal tissue has been interpreted as an ‘ostiole’ (e.g., Fig. 2b) when it appears more likely to be tissue loss by damage. Distinct ostioles appear in perhaps eight taxa. Twelve taxa, including *H. eboracense*, *P. doratophylli* and *A. dictozamiticola,* were collected on cycadophyte cuticles. With two possible exceptions (*Notothyrites haizhouensis* and *Perisporiacites zamiophylli*) the fossils on cycadophytes, *H. eboracense* included, lacked a distinct ostiole or slit-like openings for spore release.

Characters of the Mesozoic fossils, including *H. eboracense* (Table 1), are found in *Leotiomyceta* (Eriksson and Winka 1997), a superclass in *Pezizomycotina*, *Ascomycota* (Fig. 3, Additional file 1: Table S1). *Leotiomyceta* represents one of two basal clades in *Pezizomycotina*. Surveying published data (Additional file 1: Table S1) revealed that leaf-associated, melanized stromata are widely distributed across *Leotiomyceta* classes (Fig. 3). Such stromata are unknown in the sister group to *Leotiomyceta*, a clade comprising classes *Pezizomycetes* and *Orbiliomycetes* (Fig. 3). Leaf-associated, melanized stromata are likewise unknown in other *Ascomycota* subphyla, *Saccharomycotina* or *Taphrinomycotina* (Fig. 3).

Further, the epicuticular initiation of sporomata or stromata of any kind is rare or absent in orders outside of *Pezizomycotina* (Fig. 3, Additional file 1: Table S1), whereas fungi in *Pezizomycotina* present examples of all sporomatal types in *Ascomycota* (apothecioid, perithecioid, thyriothecioid, cleistothecioid) in association with plant leaves. Out of 89 extant taxa surveyed, stromata of radiating hyphae occur in lineages spanning the diversity of *Leotiomyceta*: *Arthoniomycetes*, *Dothideomycetes*, *Eurotiomycetes*, *Lecanoromycetes,* and *Sordariomycetes* (Fig. 3, Additional file 1: Table S1) and include thyriothecioid, catathecioid, apothecioid, and perithecioid sporomata, as well as some asexual stromata. Radiate thyriothecia with a well-defined central ostiole (sometimes with robust setae around the opening) or with radial slits for spore release are known from extant *Dothideomycetes* (Fig. 3, Table 1, Additional file 1: Table S1).

## PALEOGEOGRAPHY

Reconstructing the paleolatitude of the fossil-bearing localities shows that most localities are distributed between 31.5 and 60.5 absolute degrees (Fig. 4a), the only exception being the Guaduas locality from the Maastrichtian of Colombia (2.9 degrees north). The totality of the plant-bearing localities, although showing an enrichment at high-latitude localities especially during the Jurassic, show a much more regular distribution of localities across latitudes (Fig. 4a). The Mesozoic localities including the Jurassic Gristhorpe (UK), Daohugou (China), and Irkutsk (Russia) localities and the Early Cretaceous Razdol’naya depression (Russia), Shahezi (China), Sivaganga (India), Patuxtent formation (USA), Longarm formation (Canada) and English Wealden (UK) localities share floristic and paleobiogeographic similarities. The two Jurassic localities share a similar paleolatitude (43.3° N for the Gristhorpe plant bed and 43.6° N for the Daohugou) (Fig. 4b) as well as a similar floristic composition including *Bennettitales*, ginkgophytes, ferns, and seed ferns. Cycads are absent in the Daohugou locality while they are rather diverse in the Gristhorpe. Moreover, the two localities differ in the relative diversity of each group, with fewer fern taxa; more ginkgophytes and some lycophytes and horsetails are present in the Daohugou locality. The Irkutsk flora, although found at higher latitudes (60.5° N), is part of the same phytogeographical province as the Daohugou locality (Kiritchkova et al. 2022).

The Early Cretaceous localities are also of similar paleolatitudes (Potomac ~ 31° N, Wealden ~ 41° N, Longarm ~ 36° N, Shahezi ~ 48° N, Razdol’naya depression ~ 50° Sivaganga ~ 50° S) (Fig. 4a, b). Though they represent a significant timespan (Hauterivian for the English Wealden, Aptian for Shahezi, Sivaganga,—and for the Patuxtent formation, Aptian–Albian for the Razdol’naya depression).

With the exception of the Longarm formation, which is marine and includes plant remains only as permineralized nodules thus precluding a clear floristic comparison with the other localities, the Early Cretaceous localities share the presence of abundant ferns, *Bennettitales*, broad-leaved conifers, and seed ferns, with the cycads *Nilssonia* and *Ctenis* present in Northern Hemisphere localities. Although it is difficult to infer local assemblages based on the taphocoenoses of localities with different depositional histories, the overall composition of the flora is suggestive of warm temperate biomes sensu (Rees et al. 2000), with a lack of strong precipitation, seasonality or aridity.

The floras of the Late Cretaceous differ from the others in the presence and/or higher abundance of angiosperms. Moreover, *Cycadales* and *Bennettitales* are much rarer if not entirely absent.

Pie charts (Fig. 4b) represent the presence of the different fungal structures and the proportional number of species of each type.

## DISCUSSION

### Phylogenetic interpretation of leaf-associated fungal fossils and implications for the ages of appearance of ascomycete clades

Results from comparing *H. eboracense* with other extant and fossil taxa with melanized, leaf-associated stromata of radiating hyphae provide a window into the Mesozoic record of the origin and diversification of *Ascomycota.* They have particular implications for the rise of the *Leotiomyceta*, for the origins of classes *Dothideomycetes* and *Sordariomycetes*, and for the evolutionary origins of all lineages that are older than these based on phylogenomic analysis (Li et al. 2021).

We had to contend with limits to precision of phylogenetic placements of Mesozoic fossils resulting from their imperfect preservation and from the absence of information about their reproductive methods. When Mesozoic leaf-associated fungi were described but not formally named, it was usually because their structures were difficult or impossible to interpret (Harris 1961; Pons and Boureau 1977; Bajpai and Maheshwari 1987; Van der Ham and Dortangs 2005; Hübers et al. 2011; Sun et al. 2015). Phylogenies of living taxa are improving as they draw on increasingly rich samples of genes and species (Li et al. 2021). However, no matter how strong the branch support in a tree, much uncertainty remains in reconstructions of ancestral character states of extant taxa, as shown in Appendix S4 in Le Renard et al. (2020a).

Caveats aside, comparisons of fossils in the context of phylogenies of extant taxa provide the best—indeed the only—approach to tracking fungal evolution through geological time, and leaf associated fungal fossils have particular value in documenting evolution of *Ascomycota*.

### Palaeozoic *fungi*

The oldest evidence of Palaeozoic superficial fungal growths on leaf surfaces comes from the dispersed cuticle of a putative pteridosperm from the Lower Carboniferous (Middle Visean, *ca* 338 Mya) of Germany (Hübers et al. 2011). We are unable to provide a phylogenetic interpretation for this fossil beyond that it appears to be fungal. It consists of three flat hyphal structures, made of aseptate hyphae branching dichotomously from a single central area, laterally appressed to one another, forming fan-shaped lobes. Although the morphology is not sufficient to pinpoint its affinity, it resembles subcuticular infection structures described as ‘mycélium en palmettes’ on modern leaves (Ducomet 1907; Langeron 1945). The subcuticular localization suggests that the fan-shaped hyphae may have been somatic structures absorbing nutrients from its host plant.

Bajpai and Maheshwari (1987) illustrated radiate ‘discoid stromata’ from Early Permian (299–273 Mya) on *Glossopteris* leaves. The outlines of the stromata and their radially arranged branches are preserved, possibly as impressions on leaf cuticle, with little remaining of actual hyphal tissue. Whether they were melanized is uncertain, but their radiate, leaf-associated stromata, appressed to, or perhaps lying under the cuticle, are consistent with membership in *Leotiomyceta*. Because no dehiscence mechanism is evident, the stromata could represent various structures from different classes in *Leotiomyceta,* such as immature thyriothecia of *Dothideomycetes*, radiating mycelial mats as produced by some *Chaetothyriales* in *Eurotiomycetes* (Chomnunti et al. 2012), or pycnothyria as in extant *Tubakia dryina* (Holdenrieder and Kowalski 1989) (*Diaporthales*, *Sordariomycetes*).

### Mesozoic *fungi*

#### Jurassic *fungi*

The characters of *H. eboracense*, and of *Notothyrites* sp. 1 (Frolov 2018), with radial, melanized, epiphyllous stromata appressed to cuticles are consistent with membership in *Leotiomyceta*. Fossils of *Notothyrites* sp. 1 have a central opening and radial cracks but these may not be ostioles or preformed slits for dehiscence because they are irregular in shape or extent, suggesting that they resulted from physical damage rather than biological differentiation. Stromata of the Jurassic *Notothyrites* sp. 1 formed above host stomata, possibly after mycelia absorbed nutrients from internal tissues of the host. Additional taxa illustrated by Frolov include larger thalli (to 450 µm diam) located above (*Notothyrites* sp. 2) or below (*Notothyrites* sp. 3) the host cuticles, without opening mechanisms illustrated for these forms.

A leaf compression of *Sphenobaiera* from the Middle Jurassic of China (170–157 Mya) yielded flat and circular fungal structures (Sun et al. 2015). Each stroma was made up of a pseudoparenchymatous central area from which infrequently septate hyphae radiated outward. These stromata have no obvious counterparts among fossil or extant taxa.

#### Early Cretaceous *fungi*

Many of the Early Cretaceous fossils on dispersed plant cuticles are as fragmentary or difficult to interpret as the Jurassic fossils. Their hyphal morphology varies, as illustrated by Krassilov’s (1967) survey of fungal stromata from cuticles of cycadophytes and coniferophytes from the Early Cretaceous Razdol’naya River Basin in Siberia (121.4–100.5 Mya). Krassilov’s new species * Microthyriacites cephalotaxi* and *Phragmothyrites nilssonioptericola* exhibited isotomous branching hyphae leading to radial alignments (Krassilov 1967), a characteristic unknown outside of *Leotiomyceta* but found in several lineages in *Dothideomycetes*. Some of these fossils may be, as described by Krassilov (1967), thyriothecioid sporomata. Bajpai and Maheshwari (1987) also illustrated well-preserved radiate ‘discoid stromata’; again, uncertainty results from a lack of a well-defined dehiscence mechanism in these fossils. We agree with Frolov (2018) in interpreting central areas of missing tissue in the fossils as the result of damage rather than the presence of ostioles, in the absence of evidence of differentiated cells around the openings as seen in ostiolate thyriothecioid taxa (eg *Discopycnothyrium*, *Lichenopeltella*, *Microthyrium*). Further information on development or dehiscence would be needed to confirm that the fossils represent *Dothideomycetes* and not other lineages of *Leotiomyceta*.

Other Early Cretaceous (121–100.5 Mya) stromata on plant cuticles share too few characters with extant taxa to be classified and are not included in Table 1. These include *Brefeldiellites argentinus* possibly a mycelial mat of radiating cells surrounding opaque areas that may be stromata, and *Microthyriacites baqueroensis*, poorly preserved but appearing larger than other early stromata (1–1.2 mm diam), and lacking a mechanism of dehiscence, the periphery is possibly composed of radiating hyphae (Martínez 1968). *Liaoningnema multinoda* (Zheng and Zhang 1986) from the early Cretaceous consists only of hyphae.

In contrast to the ambiguity about classification of the epicuticular fossils discussed above, a permineralized fungus from the Early Cretaceous of Vancouver Island (136 Mya) in Canada provides the earliest evidence for *Sordariomycetes* (Bronson et al. 2013) and for diversification of *Pezizomycotina* crown lineages. Colonizing a coniferous leaf, *Spataporthe taylorii* produced immersed, perithecioid stromata filled with asci, a papillate ostiole, and an ostiolar canal lined with specialized hyphae called periphyses. After eliminating affinities with other classes of *Ascomycota*, Bronson et al. (2013) assigned *S. taylorii* to *Sordariomycetes*, order *Diaporthales*. However, some taxa in *Halosphaeriales* (Sakayaroj et al. 2011) and *Xylariales* (Voglmayr et al. 2018) share the combination of characters observed in *S. taylorii*. While the ordinal placement of the dehiscing sporomata of *S. taylorii* can be debated, the fossil marks the minimum age of major lineages of *Pezizomycotina*. If *Sordariomycetes* existed, so did its sister lineage, the class *Leotiomycetes* (based on the phylogeny in Fig. 3). The clade comprising *Dothideomycetes* and *Lecanoromycetes*, must have evolved earlier. Although not represented by fossils, *Pezizomycetes* and *Orbiliomycetes* or their stem lineage must also have evolved before the appearance of *Sordariomycetes*.

*Stomiopeltites cretaceous* (Alvin and Muir 1970), from the Wealden Group of England (129–121.4 Mya, Hauterivian) from dispersed cuticle is similar to some ostiolate thyriothecia produced by extant taxa such as species of *Stomiopeltis* (*Dothideomycetes*) or *Micropeltis* (*Lecanoromycetes*) (Le Renard et al. 2020a). Alvin and Muir’s (1970) scanning electron micrographs reveal the stromata to be a scutellum made of a dense network of tightly intertwined hyphae sitting on the cuticle. Supporting interpretation as a thyriothecium, rather than a catathecium or perithecium, is the apparent lack of a lower wall when viewed through the ostiole (Pl.1F in Alvin and Muir 1970). Thus, although the class-level position is uncertain, *S. cretaceus* is another fossil pointing to an Early Cretaceous diversification of *Leotiomyceta*.

Convincing evidence of early *Dothideomycetes* comes from morphological and phylogenetic analysis of fungal fossils from the Early Cretaceous (121.4–113 Mya, Aptian) cuticles of conifers from the Potomac Group beds (Patuxent formation) (Le Renard et al. 2020b, 2021a, b). Development of thyriothecioid stromata from multiple generative hyphae and branching through pseudomonopodial dichotomies is consistent with a classification of *Protographum luttrellii* in *Dothideomycetes*, possibly near order *Lembosinales* (Le Renard et al. 2020b). *Bleximothyrium ostiolatum* produced radiate, ostiolate stromata, appressoria, penetration pegs, and an extensive subcuticular mycelium, resolving its position as among *Dothideomycetes* (Le Renard et al. 2021b). The radiate stromata of *Stomatothyrium placocentrum* can be placed in *Dothideomycetes* because they are appressed to the leaf and apparently rupture by circular slits in the scutellum (Le Renard et al. 2021a).

Early Cretaceous Shahezi Formation (121.4–113 Mya, Aptian) fossils *Microthyriacites fuxinensis*, *M. plicatus*, and * Notothyrites haizhouensis* all have epicuticular stomata of regular, radially aligned hyphae but without a dehiscence mechanism (Zheng and Zhang 1986). No clues about their early development are available but their morphology is consistent with a placement in *Leotiomyceta*, *Dothideomycetes,* or possibly *Sordariomycetes*.

#### Late Cretaceous *fungi*

Pons and Boureau (1977) described a probable *Dothideomycetes* fossil, *Mariusia andegavensis*, from the Late Cretaceous of Anjou in France (100–94 Mya, Cenomanian). Found on the cuticles of *Cheirolepidiaceae*, the fossil has ostiolate, thyriothecioid stromata and an extensive network of superficial and subcuticular hyphae. The thyriothecioid stromata are similar to * Bleximothyrium ostiolatum,* but a radial pattern of scutellum hyphae is difficult to trace in *M. andegavensis* due to the interweaving of the hyphal filaments. Like *B. ostiolatum*, the apparatus for nutrient uptake in *M. andegavensis* consists of extensive, superficial appressoria with penetration pegs and subcuticular ‘mycélium en palmettes’ (Pons and Boureau 1977). Characters of the hyphae of *B. ostiolatum* and *M. andegavensis* are similar to some modern *Venturiales* and *Microthyriales* (Le Renard et al. 2021b). However, details about the taxonomic distributions of appressoria and ‘mycélium en palmettes’ in living taxa remain incomplete, limiting their usefulness in classification of fossils.

The fossil * Callimothallus corralesense*, from the Late Cretaceous of Colombia (72–66 Mya, Maastrichtian), produced radiate stromata on angiosperm leaves (Doubinger and Pons 1975). Characterized by cells with a minute pore, *Callimothallus* is hypothesized to represent sporodochia of the order *Muyocopronales* in *Dothideomycetes* (Hernández-Restrepo et al. 2019; Worobiec et al. 2020). Taxa in *Muyocopronales* produce asexual spores from each of the minute pores that decorate the dorsal surface of cells in the stroma.

Van der Ham and Dortangs (2005) described two taxa in cross section from silicified leaves of *Brachyphyllum patens* from the Lower Maastricht Formation (72–66 Mya, Maastrichtian). *Pteropus brachyphylli* produced stromata on the plant surface arising from the host stomata and was interpreted as a member of the order *Venturiales* in *Dothideomycetes* by analogy with stromata of *Phaeocryptopus gaeumannii*. However, several *Dothideomycetes* lineages in addition to *Venturiales* produce stromata through stomata (Guatimosim et al. 2014; Groenewald et al. 2013), and some taxa in *Leotiomycetes* also form extensive stromata that regularly extend below stomata (Minter 1993; Jewell 1986). For this reason, affinities cannot be inferred from stromatal characters alone (Le Renard et al. 2021a; Pons and Boureau 1977).

The cross section illustrating the other fungus, labelled ‘epicuticular stroma’, shows what appears to be a classical thyriothecium with an ostiolate, dorsal scutellum and without a basal cell wall (Van der Ham and Dortangs 2005). Without a surface view of the stroma however, the patterns of hyphal organization cannot be seen for comparison with fossils from compressions or dispersed plant cuticles.

Two additional species of fossilized fungi described from permineralized stromata from the Late Cretaceous can only be interpreted as probable members of *Leotiomyceta*. *Xylomites cycadeoideae* (100–94 Mya, Cenomanian) of New Jersey, USA, produces irregular stromata of melanized pseudoparenchyma and hyphae located below and above the plant epidermis. *Pleosporites shirainus*, from the Late Cretaceous (93.9–83.6, Mya Turonian–Santonian) (Nishida, 1991) of Japan was immersed in the epidermis of a coniferous shoot (Suzuki 1910). Described as ostiolate and producing asci, this fungus may represent a perithecioid sporoma, but the published photographs are insufficient to support this interpretation and more study of the original slides would be valuable.

#### Appearance and distribution through time of the leaf-associated *fungi*

The Gristhorpe flora has evidence of leaf-associated fungi growing on either ferns or cycadophytes, while the Daohugou fungi grew on ginkgophytes (Sun et al. 2015). The Early Cretaceous Shahezi, Razdol’naya, and Sivaganga localities also show fungi colonizing cycadophytic foliage: *Ctenis* and *Nilssonia* in the Shahezi (Zheng and Zhang 1986), *Doratophyllum*, *Nilssoniopteris*, *Dictyozamites*, and *Otozamites* in the Razdol’naya depression (Krassilov 1967), and *Ctenozamites* in the Sivaganga (Bajpai and Maheshwari 1987). These observations show that broad leaved gymnosperms (especially cycadophytes) growing in warm temperate wet forest might have been the first environment for the radiation of leaf-associated fungi in *Leotiomycetes*. The spread of the angiosperms (Coiro et al. 2019) and their effect on the vegetation structure and biome composition perhaps provided new opportunities for fungi. Indeed, equatorial floras in Colombia from the uppermost Cretaceous (76–66 Mya, Maastrichtian) (Fig. 4d) that present a rich angiosperm flora also present traces of leaf-associated fungi (Doubinger and Pons 1970, 1975), while the other, younger Late Cretaceous localities (Anjou, Raritan, Hokkaido, Romontbos) contain only fungi associated with gymnospermous fossils (Table 1). Further expansion of leaf-associated *Dothideomycetes* fungi into tropical and equatorial environments appears to have followed the Cretaceous–Paleogene (66 Mya) extinction (Carvalho et al. 2021) and continues to the present (Le Renard et al. 2020a).

## CONCLUSION

In this study, we described *Harristroma eboracense*, a new fossil leaf-associated fungus from the Jurassic (*ca* 170 Mya). The advance of molecular phylogenetics offers an opportunity to relate characters of this and other ancient fossils to clades of extant taxa. Our comparisons of modern with fossilized fungi are consistent with assigning *H. eboracense* and other leaf-associated, radiate, fungal stromata to the ascomycete clade *Leotiomyceta*. The oldest fossilized dehiscent sporomata, from the Early Cretaceous, are taken to represent *Sordariomycetes* and *Dothideomycetes*. If these interpretations are correct, these epicuticular fossils give a minimum age of Early Permian, 273 Mya on the first divergence of crown *Pezizomycotina*, and a minimum age of the Early Cretaceous, 121.4 Mya on the first divergences of crown *Leotiomyceta* leading to the establishment of classes *Dothideomycetes* and *Sordariomycetes*. The study also shows that warm temperate wet forests might have been the first environment for the radiation of *Leotiomyceta.* Moreover, the wide distribution of leaf-associated fungi during the Jurassic and Early Cretaceous suggests that macroclimatic or floristic conditions rather than geographical barriers determined the distribution of these fungi.

## Supplementary Information

Additional file1 .

## Data Availability

The data used in this manuscript are available in the text and figures, and the data used for paleobiogeography are available on Figshare: 10.6084/m9.figshare.24417955.
