# Supplementary material for: A new leaf inhabiting ascomycete from the Jurassic (ca 170 Mya) of Yorkshire, UK, and insights into the appearance and diversification of filamentous Ascomycota
Source: IMA Fungus. 2024 Nov 5;15:34. doi: 10.1186/s43008-024-00162-9 (PMC11536623; doi:10.1186/s43008-024-00162-9)
Supplement: Supplementary file 1 — Additional file1 . [file 43008_2024_162_MOESM1_ESM.docx]

**Supplementary Data**

**Table S1.** Sporocarp characters of Ascomycota included in the phylogeny of Li *et al.*, (2021) and additional foliicolous fungi.

Taxa sharing characters with *Harristroma* *eboracense* sp. nov. are indicated in bold.

|  | **Taxon / Reference culture or GenBank accession** | **Sporoma type (as described)** | | **Radiate superficial growth (details)** | | **M** | | **F** | **Reference and illustration(s)** |
| --- | --- | --- | --- | --- | --- | --- | --- | --- | --- |
| Fossil | | | | | | | | | |
| *Harristroma eboracense* gen. et. sp. nov. | | | Unknown | Yes | Yes | | Yes | | Fig. 1 |
| Pezizomycotina | | | | | | | | | |
| Arthoniomycetes | | | | | | | | | |
| Arthoniales | | | | | | | | | |
|  | *Arthonia radiata* / EZ20314 | Apothecioid (apothecia) | | unknown | | Yes | | No | (Acharius 1808)Not illustrated |
| *** | *Roccella fuciformis* / AFTOL-ID 126 | Apothecioid (apothecia) | | unknown | | ? | | No | (Tehler et al. 2004) |
| Lichenostigmatales | | | | | | | | | |
| *** | *Lichenostigma gracilis* / None | Cleistothecioid (stroma, non-ostiolate) | | Yes (infection begins with radiate vegetative hyphae) | | Yes | | No | (Calatayud et al. 2002) Figs. 19–27, 47 |
| Dothideomycetes | | | | | | | | | |
| Asterinales | | | | | | | | | |
| ******* | ***Asterina melastomatis* / None** | **Thyriothecioid** | | **Yes** | | **Yes** | | **Yes** | **(Guatimosim et al. 2015) Fig. 3** |
| Botryosphaeriales | | | | | | | | | |
|  | *Botryosphaeria dothidea* / LW030101 | Perithecioid (ostiolate ascomata) | | No | | Yes | | No | (Slippers et al. 2004) Figs. 1–7 |
|  | *Phyllosticta capitalensis* / CBS 173.77 | Perithecioid (ostiolate ascoma, pycnidia) | | Unknown | | Yes | | Yes | (Liu et al. 2012) Fig. 31 |
|  |  |  |  |  |  |  |  |  |  |
| Capnodiales | | | | | | | | | |
|  | *Dothistroma pini* / CBS 116487 | Asexual exposed sporoma (acervuli) | | No | | Yes | | Yes | (Hulbary 1941) Figs. 1–3 |
|  | *Leptoxyphium fumago* CBS 123.26 | Perithecioid (pycnidia) | | No | | Yes | | Yes | (Srivastava 1982) Figs. 4–7 |
|  | *Mycosphaerella eumusae* / CBS 114824 | Perithecioid (ostiolate pseudostroma) | | No | | Yes | | Yes | (Crous & Mourichon 2002) Figs. 1–12 |
|  | *Sphaerulina musiva* / SO2202 | Perithecioid (perithecia, pycnidia) | | Unknown | | Yes | | Yes | (Bier 1939) Pls. I–V |
| Dothideales | | | | | | | | | |
|  | *Aureobadisium subglaciale* / EXF-2481 | Unknown | | NA | | NA | | No | (Gostinčar et al. 2014) Fig. 1 |
| Hysteriales | | | | | | | | | |
|  | *Hysterium pulicare* / CBS 123377 | Apothecioid or perithecioid (hysterothecia) | | Unknown | | Yes | | No | [link](http://www.ascofrance.com/recolte/553/dothideomycetes-hysteriales-hysteriaceae-hysterium-pulicare) |
|  | *Rhytidhysteron rufulum* / GQ221893 | Apothecioid or perithecioid (hysterothecia) | | Unknown | | Yes | | No | (Bezerra & Kimbrough 1982) Figs. 1–52 |
| *** | *Ericboehmia saulensis* / NG_075302 | Apothecioid or perithecioid (hysterothecia) | | Unknown | | Yes | | No | (Gardiennet et al. 2019) Fig. 1 |
| Lembosinales | | | | | | | | | |
| ******* | ***Lembosina aulographoides* / CBS 145946** | **Thyriothecioid (thyriothecia)** | | **Yes** | | **Yes** | | **Yes** | **(Le Renard et al. 2020b) Fig.2 B, C, E, F, S** |
| Microthyriales | | | | | | | | | |
| ***** | **Microthyrium macrosporum / CBS 143810** | **Thyriothecioid (thyriothecia)** | | **Yes** | | **Yes** | | **Yes** | **(Le Renard et al. 2020a) Fig. 4 B–F** |
| Muyocopronales | | | | | | | | | |
| ***** | **Muyocopron dipterocarpi / MFLUCC 14-1103** | **Thyriothecioid (thyriothecia, sporodochia)** | | **Yes** | | **Yes** | | **Yes** | **(Mapook et al. 2016) Fig. 3** |
| Myriangiales | | | | | | | | | |
|  | *Elsinoe australis* / NL1 | Apothecioid? (“ascomata devoid of a well-defined epithecium”, acervulus) | | No | | Yes | | No | (Bitancourt & Jenkins 1936) Figs. 1–2 |
|  | *Sphaceloma murrayae* / CQ-2017a | Asexual sporoma (acervuli) | | No | | Yes | | Yes | (Butin & Kehr 2004) Figs. 1–3 |
| Mytilinidiales | | | | | | | | | |
|  | *Lepidopterella palustris* / CBS 459.81 | Cleistothecioid (non-ostiolate multilayered ascocarp) | | No | | Yes | | No | (Shearer & Crane 1980) Figs. 7, 8, 13–28 |
| ******* | ***Mytilinidion scolecosporum* / CBS 305.34** | **Apothecioid or Perithecioid (conchiform hysterothecia)** | | **Yes (raised up sporocarp walls made of radiating hyphae)** | | **Yes** | | **Yes** | **(Lohman 1932) Fig. 1C** |
| Pleosporales | | | | | | | | | |
|  | *Beverwykella pulmonaria* / JCM 9230 | None (hyphomycete) | | No | | NA | | No | (Seifert et al. 2011) Pl. 373A |
|  | *Alternaria alternata* / B2a | None (hyphomycete) | | Unknown | | NA | | Yes | (Woudenberg et al. 2013) Fig. 4 C, H–J |
|  | *Bipolaris maydis* / ATCC 48331 | Perithecioid (ostiolate ascoma) | | Unknown | | Yes | | Yes | (Manamgoda et al. 2014) Fig. 22 |
|  | *Pyrenophora tritici-repentis* / DAOM 197047 | Perithecioid (ostiolate pseudostroma) | | No | | Yes | | Yes | (Wehmeyer 1954) Figs. 1–52 |
| *** | *Massaria gigantispora* / CBS 125593 | Perithecioid (ostiolate pseudostroma) | | No | | Yes | | No | (Voglmayr & Jaklitsch 2011)Fig. 9 |
| *** | *Phoma billsii* / ATCC MYA3680 | Perithecioid (pycnidia) | | No | | Yes | | Yes | (Torres et al. 2005) Figs. 1–9 |
| Venturiales | | | | | | | | | |
|  | *Ochroconis constricta* / UM 578 | Unknown | | No | | ? | | No | (Hoog & Arx 1974) Fig. 1 d–e |
|  | *Venturia pyrina* / ICMP 11032 | Perithecioid (ostiolate ascoma) | | No | | Yes | | Yes | (Hyde et al. 2013) Fig. 131 |
|  | *Verruconis gallopava* / | Unknown | | No | | ? | | No | (Grewal et al. 2018) Fig. 1 |
|  | CBS 43764 |  |  |  |  |  |  |  |  |
|  | *Glonium stellatum* / CBS 207.34 | Perithecioid or Apothecioid? (ascoma opening by longitudinal slits) | | No | | Yes | | No | (Luttrell 1953) Figs. 1–21 |
| Incertae sedis (Dothideomycetes) | | | | | | | | | |
| ******* | *Peltaster fructicola* / LNHT1506 | Thyriothecioid (pycnothyria) | | Yes | | Yes | | No | (Williamson et al. 2004) Figs. 5–8 |
| ******* | *Catinella olivacea*/ UAMH 10679 | Apothecioid (apothecia) | | Yes (‘anchoring hyphae’ radiating from the base of the excipulum) | | Yes | | No | (Greif et al. 2007) Figs. 1–27 |
| *** | *Rhizodiscina lignyota* | Apothecioid (apothecia) | | No | | ? | | No | (Uzun & Kaya 2018) Figs. 1–2 |
| Eurotiomycetes | | | | | | | | | |
| Chaetothyriales | | | | | | | | | |
|  | *Capronia coronata* / CBS 617.96 | Perithecioid (ostiolate ascoma) | | Unknown | | Yes | | No | (Müller et al. 1987) Figs. 3, 4I |
|  | *Exophiala calicioides* / JCM 6030 | Unknown | | Unknown | | ? | | No | (Ellis 1971) Fig. 229 B |
| *** | ***Ceramothyrium thailandicum* / MFLUCC 10–0079** | **Perithecioid (non-ostiolate ascoma)** | | **Yes (radiating mycelial mat)** | | **Yes** | | **Yes** | **(Chomnunti et al. 2012) Fig. 3** |
| Eurotiales | | | | | | | | | |
|  | *Elaphomyces granulatus* / OSC145934 | Cleistothecioid (cleistothecia) | | Unknown | | Yes | | No | (Geiser 2009) Table 1 |
|  | *Aspergillus nidulans* ATCC 10074 | Cleistothecioid (cleistothecia) | | No | | Yes | | No | (Geiser 2009) Table 1 |
|  | *Penicillium chrysogenum* / HKF 42 | Cleistothecioid (cleistothecia) | | No | | No | | No | (Böhm et al. 2013) Fig. 1A |
|  | *Xeromyces bisporus* / FRR 0525 | Cleistothecioid (cleistothecia) | | No | | No | | No | (Pettersson et al. 2011) Figs. 4–6 |
| Phaeomoniellales | | | | | | | | | |
|  | *Phaeomoniella chlamydospora* / UCRPC4 | Perithecioid (Phoma-like synanamorph) | | No | | Yes | | No | (Gams & Crous 2000) Figs. 6-8 |
| Verrucariales | | | | | | | | | |
|  | *Endocarpon pusillum* / KoLRI No. LF000583 | Perithecioid (ostiolate ascoma) | | Unknown | | Yes | | No | (Bertsch & Butin 1966) Figs. 1–7 |
| Sclerococcales | | | | | | | | | |
| * | *Pseudosclerococcum golindoi* / CBS 143732 | Apothecioid (apothecia) | | Unknown | | Yes | | No | (Olariaga et al. 2019) Figs. 2–3 |
| Lecanoromycetes | | | | | | | | | |
| Lecanorales | | | | | | | | | |
|  | *Cladonia macilenta* / KoLRI003786 | Apothecioid (apothecia) | | Unknown | | No | | No | (Fink 1907) Fig. 2 |
|  | *Evernia prunastri* / FR SP7-11 | Apothecioid (apothecia) | | Unknown | | No | | No | (Howe Jr 1911) Fig. 1 |
|  | *Ramalina intermedia* / YAF0013 | Apothecioid (apothecia) | | Unknown | | No | | No | (Bowler & Rundel 1974) |
| Ostropales | | | | | | | | | |
| *** | *Acarosporina microspora* / AFTOL-ID 78 | Apothecioid and perithecioid (apothecia and pycnidia) | | Unknown | | Yes | | No | (Sherwood 1977) p. 36 |
| *** | *Porina epiphylla*  / AFTOL-ID 343 | Perithecioid (perithecia) | | Unknown | | Yes | | Yes | (Lücking 2008) Figs. 128E, 129E |
| *** | *Asterothyrium leucophtalmum* / isolate 23081 | Apothecioid and perithecioid (apothecia and pycnidia) | | Yes | | No | | Yes | (Henssen & Lücking 2002) Fig. 9 |
| * | *Micropeltis zingiberacicola* / IFRDCC 2264 | Thyriothecioid (thyriothecia) | | No | | Yes | | Yes | (Wu et al. 2011) Fig. 11 |
| Teloschistales | | | | | | | | | |
|  | *Gyalolechia flavorubescens* / KoLRI002931 | Apothecioid (apothecia) | | No | | No | | No | [link](https://lichenportal.org/cnalh/taxa/index.php?taxauthid=1&taxon=56259&clid=1069) |
| Umbilicariales | | | | | | | | | |
|  | *Umbilicaria muehlenbergii* / KoLRI No. LF000956 | Apothecioid (apothecia) | | No | | Yes? | | No | (Himelbrant 2016) |
| Leotiomycetes | | | | | | | | | |
| Erisyphales | | | | | | | | | |
|  | *Blumeria graminis* f. sp. *tritici* / 96224 | Cleistothecioid (chasmothecia) | | No | | Yes | | Yes | (Jankovics et al. 2015) Figs. 2–6 |
|  | *Erysiphe necator* / C-strain | Cleistothecioid (chasmothecia) | | No | | Yes | | Yes | (Bendezú-Euribe & Alvarez 2012) Supplementary figures 1–2 |
| Helotiales | | | | | | | | | |
|  | *Coleophoma cylindrospora* / CBS 591.70 | Perithecioid (pycnidia) | | No | | Yes | | Yes | (Crous & Groenewald) Fig. 8 |
|  | *Hymenoscyphus fraxineus* / CBS 133217 | Apothecioid (apothecia) | | Unknown | | No | | Yes | (Han et al. 2014) Figs. 1–3 |
|  | *Lanzia echinophila* / CBS 111548 | Apothecioid (apothecia) | | Unknown | | No | | No | (van der Nest et al. 2014) Fig. 2A–D |
| Orbiliomycetes | | | | | | | | | |
| Orbiliales | | | | | | | | | |
|  | *Dactylellina haptotyla* / CBS 200.50 | Unknown | | NA | | No | | No | (Baral et al. 2020) Fig. 103i |
|  | *Orbilia quercus* / HMAS 88783 | Apothecioid (apothecia) | | No | | No | | No | (Liu et al. 2005) Figs. 1–2 |
| Pezizomycetes | | | | | | | | | |
| Pezizales | | | | | | | | | |
|  | *Morchella eximia* / MG90 | Apothecioid (apothecia) | | No | | No | | No | (Perić & Radić 2013) Figs. 8–11 |
|  | *Pyronema omphalodes* / CBS100304 | Apothecioid (apothecia) | | No | | No | | No | [link](http://www.ascofrance.com/recolte/2731/pezizomycetes-pezizales-pyronemataceae-pyronema-omphalodes) |
|  | *Tuber melanosporum* / Mel28 | Apothecioid | | No | | No | | No | [link](http://www.ascofrance.com/recolte/1227/pezizomycetes-pezizales-tuberaceae-tuber-melanosporum) |
| Sordariomycetes | | | | | | | | | |
| Amphisphaeriales | | | | | | | | | |
| * | *Pestalotiopsis gibbosa* / NOF3175 | Apothecioid (apothecia, acervuli) | | No | | Yes | | Yes | (Watanabe et al. 2018) Figs. 1, 3 |
| * | *Pestalotiopsis microspora* / none | Perithecioid (perithecia, acervuli) | | No | | Yes | | ? | (Metz et al. 2000) Figs. 1–3 |
| Coniochaetales | | | | | | | | | |
|  | *Coniochaeta ligniaria* / NRRL 30616 | Perithecioid | | Unknown | | Yes | | No | [link](http://www.ascofrance.com/recolte/4060/sordariomycetes-coniochaetales-coniochaetaceae-coniochaeta-ligniaria) |
| Diaporthales | | | | | | | | | |
|  | *Juglanconis oblonga* / AR4414 | Perithecioid (perithecia, acervuli) | | Unknown | | Yes | | No | (Voglmayr et al. 2017) Figs. |
| * | ***Tubakia dryina* / CBS 112097** | **Perithecioid (pycnidium, rhizothyrium)** | | **Yes** | | **Yes** | | **Yes** | **(Holdenrieder & Kowalski 1989) Figs. 1–11** |
| Halosphaeriales | | | | | | | | | |
| * | *Panorbis viscosus* | Perithecioid (perithecia) | | Unknown | | Yes | | no | [link](https://www.marinefungi.org/species_monograph/31/viscosus-/) |
| Hypocreales | | | | | | | | | |
|  | *Cordyceps militaris* / CM01 | Perithecioid (perithecia) | | No | | ? | | No | (Sung et al. 2007) Fig. 9A, 9O, 9R |
| Magnaporthales | | | | | | | | | |
|  | *Gaeumannomyces tritici* / R3-111a-1 | Perithecioid (perithecia) | | Unknown | | Yes | | Yes | (Freeman & Ward 2004) Fig. 1 |
| Meliolales | | | | | | | | | |
| * | ***Meliola mucunicola* / MFLU15-0386** | **Perithecioid (perithecia)** | | **Yes** | | **Yes** | | **Yes** | **(Hongsanan et al. 2015) Fig. 24** |
| Microascales | | | | | | | | | |
|  | Ceratocystis fimbriata CBS 114723 | Perithecioid (perithecia) | | No | | Yes | | No | (Engelbrecht & Harrington 2005) Figs. 1–3 |
| Ophiostomatales | | | | | | | | | |
|  | Ophiostoma piceae / UAMH 11346 | Perithecioid (perithecia) | | No | | Yes | | No | (Harrington et al. 2001) Figs. 2–12 |
| Sordariales | | | | | | | | | |
|  | *Chaetomium globosum* / CBS 148.51 | Perithecioid | | No | | Yes | | No | [link](http://www.ascofrance.com/recolte/3496/sordariomycetes-sordariales-chaetomiaceae-chaetomium-globosum) |
| Xylariales | | | | | | | | | |
| * | *Pestalotiopsis gibbosa* / NOF3175 | Apothecioid (apothecia, acervuli) | | No | | Yes | | Yes | (Watanabe et al. 2018) Figs. 1, 3 |
| * | *Pestalotiopsis microspora* / none | Perithecioid (perithecia, acervuli) | | No | | Yes | | ? | (Metz et al. 2000) Figs. 1–3 |
|  | *Rosellinia necatrix* / W97 | Perithecioid | | No | | Yes | | No | [link](http://www.ascofrance.com/recolte/2061/sordariomycetes-xylariales-xylariaceae-rosellinia-necatrix) |
| Xylonomycetes | | | | | | | | | |
| Symbiotaphrinales | | | | | | | | | |
|  | *Symbiotaphrina buchneri* / JCM 9740 | None | | NA | | NA | | No | (Gams & von Arx 1980) |
| Xylonales | | | | | | | | | |
|  | *Xylona heveae* TC161 / TC161 | Cleistothecioid (non-ostiolate pycnidium) | | Unknown | | No | | Yes | (Gazis et al. 2012) Fig. 3 |
| **Incertae sedis (Pezizomycotina)** | | | | | | | | | |
| ***** | *Zelosatchmopsis sacciformis* / none | Asexual sporoma (cupulate conidioma) | | Yes | | Yes | | No | (Saikawa et al. 1991) Figs. 1–9 |
| Saccharomycotina | | | | | | | | | |
| Saccharomycetes | | | | | | | | | |
| Saccharomycetales | | | | | | | | | |
|  | *Candida albicans* / SC5314 | None | | NA | | NA | | No | (Lachance et al. 2011) |
|  | *Debaryomyces hansenii* / CBS 767 | None | | NA | | NA | | No | (Suzuki et al. 2011) |
|  | Saccharomyces cerevisiae / S288C | None | | NA | | NA | | No | (Vaughan-Martini & Martini 2011a) |
| Taphrinomycotina | | | | | | | | | |
| Neolectomycetes | | | | | | | | | |
| Neolectales | | | | | | | | | |
|  | *Neolecta irregularis* /  DAH-3 | Apothecioid (apothecia) | | No | | No | | No | (Redhead 1977) Figs. 1, 3, 4, 8, 9 |
| Incertae sedis (Neolectomycetes) | | | | | | | | | |
|  | *Saitoella complicata* / NRRL Y-17804 | None | | NA | | NA | | No | (Sugiyama & Hamamoto 2011) Fig. 96.2 |
| Pneumocystidomycetes | | | | | | | | | |
| Pneumocystidiales | | | | | | | | | |
|  | *Pneumocystis jirovecii* / RU7 | None | | NA | | NA | | No | (Cushion & Keely 2011) Fig. 58.5 |
| Schizosaccharomycetes | | | | | | | | | |
| Schizosaccharomycetales | | | | | | | | | |
|  | *Schizosaccharomyces pombe* / 972h- | None | | NA | | NA | | No | (Vaughan-Martini & Martini 2011b) Figs. 66.4–66.5 |
| Taphrinomycetes | | | | | | | | | |
| Taphrinales | | | | | | | | | |
|  | *Protomyces lactucaedebilis* / 12-1054 | None | | NA | | NA | | ? | (Kurtzman 2011) |
|  | *Taphrina deformans* / JCM 22205 | None | | NA | | NA | | Yes | (Kramer 1987) Figs. 5–8 |
| * | *Taphrina maculans* | None | | Yes (radiating hyphae prior to host penetration) | | No | | Yes | (Upadhyay & Pavgi 1973) Figs. 6–9 |

Notes: Taxa absent from Li *et al.*, (2021) are indicated with *

M = produces melanized stromata

F = foliicolous

**LITERATURE CITED**

Acharius, E. 1808. Förteckning på de i Sverige våxande arter af Lafvarnas Familj. Kongliga Vetenskaps Academiens Nya Handlingar 29: 125–132.

Baral, H-O, Weber, E, and Marson, G. 2020. Monograph of Orbiliomycetes (Ascomycota) based on vital taxonomy. National Museum of Natural History Luxembourg.

Bendezú-Euribe, MV, and Alvarez, LA. 2012. The perfect stage of powdery mildew of grapevine caused by *Erysiphe* *necator* found in Peru. Plant Disease 96: 768-768.

Bertsch, A, and Butin, H. 1966. Die Kultur der Erdflechte *Endocarpon pusillum* im Labor. Planta 72: 29-42.

Bezerra, JL, and Kimbrough, JW. 1982. Culture and cytological development of *Rhytidhysterium rufulum* on citrus. Canadian Journal of Botany 60: 568–579.

Bier, J. 1939. Septoria canker of introduced and native hybrid poplars. Canadian Journal of Research 17: 195-204.

Bitancourt, AA, and Jenkins, AE. 1936. Perfect stage of the sweet orange fruit scab fungus. Mycologia 28: 489-492.

Böhm, J, Hoff, B, O’Gorman, CM, Wolfers, S, Klix, V, Binger, D, Zadra, I, Kürnsteiner, H, Pöggeler, S, Dyer, PS, et al. 2013. Sexual reproduction and mating-type–mediated strain development in the penicillin-producing fungus *Penicillium chrysogenum*. Proceedings of the National Academy of Sciences 110: 1476-1481.

Bowler, PA, and Rundel, PW. 1974. The *Ramalina intermedia* complex in North America. Bryologist 77: 617-623.

Butin, H, and Kehr, R. 2004. Sphaceloma murrayae Jenk. & Grods., a pathogen new to Europe on Salix spp. Forest Pathology 34: 27-31.

Calatayud, V, Navarro-RosinÉS, P, and Hafellner, J. 2002. A synopsis of Lichenostigma subgen. Lichenogramma (Arthoniales), with a key to the species. Mycological Research 106: 1230-1242.

Chomnunti, P, Ko, TWK, Chukeatirote, E, Hyde, KD, Cai, L, Jones, EBG, Kodsueb, R, Hassan, BA, and Chen, H. 2012. Phylogeny of Chaetothyriaceae in northern Thailand including three new species. Mycologia 104: 382–395.

Crous, PW, and Groenewald, JZ. They seldom occur alone. Fungal Biology.

Crous, PW, and Mourichon, X. 2002. *Mycosphaerella eumusae* and its anamorph *Pseudocercospora eumusae* spp. nov.: causal agent of eumusae leaf spot disease of banana. Sydowia 54: 23–34.

Cushion, MT, and Keely, SP. 2011. Chapter 58 - *Pneumocystis* Delanoë & Delanoë (1912). In The Yeasts (Fifth Edition), Kurtzman CP, Fell JW, andBoekhout T (eds), Elsevier, London: 709-717.

Ellis, MB. 1971. Dematiaceous Hyphomycetes. Kew, Commonwealth Mycological Institute.: 608 pp.

Engelbrecht, CJB, and Harrington, TC. 2005. Intersterility, morphology and taxonomy of *Ceratocystis fimbriata* on sweet potato, cacao and sycamore. Mycologia 97: 57-69.

Fink, B. 1907. Further notes on cladonias. XII. *Cladonia bacillaris, Cladonia macilenta* and *Cladonia didyma*. The Bryologist 10: 77-79.

Freeman, J, and Ward, E. 2004. *Gaeumannomyces graminis*, the take‐all fungus and its relatives. Molecular Plant Pathology 5: 235-252.

Gams, W, and Crous, PW. 2000. *Phaeomoniella chlamydospora* gen. et comb. nov., a causal organism of Petri grapevine decline and esca. Phytopathologia Mediterranea 39: 112–118.

Gams, W, and von Arx, J. 1980. Validation of *Symbiotaphrina* (Imperfetc yeasts). Persoonia-Molecular Phylogeny and Evolution of Fungi 10: 542-543.

Gardiennet, A, Lechat, C, and Fournier, J. 2019. Ericboehmia, a new genus segregated from Ostreichnion in the Hysteriaceae, with the new species E. saulensis. Ascomyceteorg 11: 171-176.

Gazis, R, Miadlikowska, J, Lutzoni, F, Arnold, AE, and Chaverri, P. 2012. Culture-based study of endophytes associated with rubber trees in Peru reveals a new class of Pezizomycotina: Xylonomycetes. Molecular Phylogenetics and Evolution 65: 294-304.

Geiser, DM. 2009. Sexual structures in *Aspergillus*: morphology, importance and genomics. Medical Mycology 47: S21-S26.

Gostinčar, C, Ohm, RA, Kogej, T, Sonjak, S, Turk, M, Zajc, J, Zalar, P, Grube, M, Sun, H, Han, J, et al. 2014. Genome sequencing of four *Aureobasidium pullulans* varieties: biotechnological potential, stress tolerance, and description of new species. BMC Genomics 15: 549–549.

Greif, MD, Gibas, CFC, Tsuneda, A, and Currah, RS. 2007. Ascoma development and phylogeny of an apothecioid Dothideomycete, *Catinella olivacea*. American Journal of Botany 94: 1890–1899.

Grewal, HK, Kumar, PN, Shah, N, and Timpone, JG. 2018. Verruconis Gallopava: Recurrent Infection in a Renal Transplant Recipient. Infectious Diseases in Clinical Practice 26.

Guatimosim, E, Firmino, A, Bezerra, J, Pereira, O, Barreto, R, and Crous, P. 2015. Towards a phylogenetic reappraisal of Parmulariaceae and Asterinaceae (Dothideomycetes). Persoonia 35: 230–241.

Han, J-G, Shrestha, B, Hosoya, T, Lee, K-H, Sung, G-H, and Shin, H-D. 2014. First report of the ash dieback pathogen *Hymenoscyphus fraxineus* in Korea. Mycobiology 42: 391-396.

Harrington, TC, McNew, D, Steimel, J, Hofstra, D, and Farrell, R. 2001. Phylogeny and taxonomy of the *Ophiostoma piceae* complex and the Dutch elm disease fungi. Mycologia 93: 111-136.

Henssen, A, and Lücking, R. 2002. Morphology, anatomy, and ontogeny in the Asterothyriaceae (Ascomycota: Ostropales), a misunderstood group of lichenized fungi. Annales Botanici Fennici 39: 273-299.

Himelbrant, DE. 2016. The lichens and allied fungi from the Leningrad Region and Saint Petersburg in the lichen herbarium of the University of Tartu. Folia Cryptogamica Estonica 53: 35-42.

Holdenrieder, O, and Kowalski, T. 1989. Pycnidial formation and pathogenicity in *Tubakia dryina*. Mycological Research 92: 166–169.

Hongsanan, S, Tian, Q, Peršoh, D, Zeng, X-Y, Hyde, DK, Chomnunti, P, Boonmee, S, Bahkali, AH, and Wen, T-C. 2015. Meliolales. Fungal Diversity 74: 91–141.

Hoog, GSd, and Arx, JAv. 1974. Revision of *Scolecobasidium* and *Pleurophragmium*. Kavaka 1: 55–60.

Howe Jr, RH. 1911. The genus *Evernia* as represented in North and Middle America. Botanical Gazette 51: 431-442.

Hulbary, RL. 1941. A needle blight of Austrian pine. Illinois Natural History Survey Bulletin; v 021, no 07.

Hyde, KD, Jones, EBG, Liu, J-K, Ariyawansa, H, Boehm, E, Boonmee, S, Braun, U, Chomnunti, P, Crous, P, Dai, D-Q, et al. 2013. Families of Dothideomycetes. Fungal Diversity 63: 1–313.

Jankovics, T, Komáromi, J, Fábián, A, Jäger, K, Vida, G, and Kiss, L. 2015. New insights into the life cycle of the wheat powdery mildew: direct observation of ascosporic infection in *Blumeria graminis* f. sp. *tritici*. Phytopathology 105: 797-804.

Kramer, C. 1987. The Taphrinales. The expanding realm of yeast-like fungi: 151-166.

Kurtzman, CP. 2011. Chapter 60 - Protomyces Unger (1833). In The Yeasts (Fifth Edition), Kurtzman CP, Fell JW, andBoekhout T (eds), Elsevier, London: 725-731.

Lachance, M-A, Boekhout, T, Scorzetti, G, Fell, JW, and Kurtzman, CP. 2011. Chapter 90 - Candida Berkhout (1923). In The Yeasts (Fifth Edition), Kurtzman CP, Fell JW, andBoekhout T (eds), Elsevier, London: 987-1278.

Le Renard, L, Firmino, AL, Pereira, OL, Stockey, RA, and Berbee, ML. 2020a. Character evolution of modern fly-speck fungi and implications for interpreting thyriothecial fossils. American Journal of Botany 107: 1–20.

Le Renard, L, Stockey, RA, Upchurch, G, and Berbee, ML. 2020b. A new epiphyllous fly-speck fungus from the Early Cretaceous Potomac group of Virginia (125–112 Ma): *Protographum luttrellii* gen. et sp. nov. Mycologia 112: 504–518.

Le Renard, L, Stockey, RA, Upchurch, G, and Berbee, ML. 2021a. Cretaceous fungal scutella from the Lower Potomac Group Zone 1: *Stomatothyrium placocentrum* gen. et sp. nov., a dothideomycete colonizer of conifer stomata. International Journal of Plant Sciences 182: 712–729.

Le Renard, L, Stockey, RA, Upchurch, G, and Berbee, ML. 2021b. Extending the fossil record for foliicolous Dothideomycetes; *Bleximothyrium ostiolatum* gen. et sp. nov. a unique fly-speck fungus from the Lower Cretaceous of Virginia, USA. American Journal of Botany 108: 129–144.

Liu, B, Liu, X-Z, and Zhuang, W-Y. 2005. *Orbilia querci* sp. nov. and its knob-forming nematophagous anamorph. FEMS Microbiology Letters 245: 99-105.

Liu, J-K, Phookamsak, R, Doilom, M, Wikee, S, Li, Y-M, Ariyawansha, H, Boonmee, S, Chomnunti, P, Dai, D-Q, Bhat, JD, et al. 2012. Towards a natural classification of Botryosphaeriales. Fungal Diversity 57: 149-210.

Lohman, ML. 1932. Three new Species of *Mytilidion* in the proposed subgenus, Lophiopsis. Mycologia 24: 477–484.

Lücking, R. 2008. Foliicolous Lichenized Fungi, Flora Neotropica Monograph. The New York Botanical Garden Press: 866.

Luttrell, ES. 1953. Development of the Ascocarp in Glonium stellatum. American Journal of Botany 40: 626-633.

Manamgoda, DS, Rossman, AY, Castlebury, LA, Crous, PW, Madrid, H, Chukeatirote, E, and Hyde, KD. 2014. The genus *Bipolaris*. Studies in Mycology 79: 221–288.

Mapook, A, Hyde, KD, Dai, D-Q, Li, J, Jones, EBG, Bahkali, AH, and Boonmee, S. 2016. Muyocopronales, ord. nov.,(Dothideomycetes, Ascomycota) and a reappraisal of *Muyocopron* species from northern Thailand. Phytotaxa 265: 225–237.

Metz, AM, Haddad, A, Worapong, J, Long, DM, Ford, EJ, Hess, WM, and Strobel, GA. 2000. Induction of the sexual stage of Pestalotiopsis microspora, a taxol-producing fungusThe GenBank accession numbers for the sequences determined in this work are: Pestalotiopsis microspora NE-32 18S rDNA, AF104356; Pestalosphaeria hansenii ATCC 48245 18S rDNA, AF242846. Microbiology 146: 2079-2089.

Müller, E, Petrini, O, Fisher, PJ, Samuels, GJ, and Rossman, AY. 1987. Taxonomy and anamorphs of the Herpotrichiellaceae with notes on generic synonymy. Transactions of the British Mycological Society 88: 63–74.

Olariaga, I, Teres, J, Martín, J, Prieto, M, and Baral, H-O. 2019. Pseudosclerococcum golindoi gen. et sp. nov., a new taxon with apothecial ascomata and a Chalara-like anamorph within the Sclerococcales (Eurotiomycetes). Mycological Progress 18: 895-905.

Perić, B, and Radić, S. 2013. Deux taxons du genre *Morchella* (Pezizales), intéressants et rares en europe: *M. steppicola* et *M. eximia*. Mycologia Montenegrina 16: 29–44.

Pettersson, OV, Leong, S-lL, Lantz, H, Rice, T, Dijksterhuis, J, Houbraken, J, Samson, RA, and Schnürer, J. 2011. Phylogeny and intraspecific variation of the extreme xerophile, *Xeromyces bisporus*. Fungal Biology 115: 1100-1111.

Redhead, SA. 1977. The genus *Neolecta* (Neolectaceae fam. nov., Lecanorales, Ascomycetes) in Canada. Canadian Journal of Botany 55: 301-306.

Saikawa, M, Ruiz, RFC, Kendrick, B, and Raj, TN. 1991. Genera coelomycetum XXVIII. *Zelosatchmopsis* anam.-gen. nov. Canadian Journal of Botany 69: 630-633.

Seifert, KA, Morgan-Jones, G, Gams, W, and Kendrick, B. 2011. The genera of hyphomycetes. CBS-KNAW Fungal Biodiversity Centre, Utrecht, Netherlands.

Shearer, CA, and Crane, JL. 1980. Taxonomy of two cleistothecial ascomycetes with papilionaceous ascospores. Transactions of the British Mycological Society 75: 193–200.

Sherwood, MA. 1977. The ostropalean fungi. Mycotaxon 5: 1–277.

Slippers, B, Crous, PW, Denman, S, Coutinho, TA, Wingfield, BD, and Wingfield, MJ. 2004. Combined multiple gene genealogies and phenotypic characters differentiate several species previously identified as *Botryosphaeria dothidea*. Mycologia 96: 83–101.

Srivastava, RC. 1982. Notes on two interesting fungi from india. Archiv für Protistenkunde 125: 331–333.

Sugiyama, J, and Hamamoto, M. 2011. Chapter 96 - Saitoella S. Goto, Sugiyama, Hamamoto & Komagata (1987). In The Yeasts (Fifth Edition), Kurtzman CP, Fell JW, andBoekhout T (eds), Elsevier, London: 1313-1315.

Sung, G-H, Hywel-Jones, NL, Sung, J-M, Luangsa-ard, JJ, Shrestha, B, and Spatafora, JW. 2007. Phylogenetic classification of *Cordyceps* and the clavicipitaceous fungi. Studies in Mycology 57: 5–59.

Suzuki, M, Prasad, GS, and Kurtzman, CP. 2011. Chapter 24 - *Debaryomyces* Lodder & Kreger-van Rij (1952). In The Yeasts (Fifth Edition), Kurtzman CP, Fell JW, andBoekhout T (eds), Elsevier, London: 361-372.

Tehler, A, Dahlkild, Å, Eldenäs, P, and Feige, GB. 2004. The phylogeny and taxonomy of Macaronesian, European and Mediterranean Roccella (Roccellaceae, Arthoniales). Symbolae Botanicae Upsalienses 34: 405-428.

Torres, MS, White Jr, JE, Cazares, G, Bergen, M, Bischoff, JE, and Sullivan, RE. 2005. A new species and its phylogenetic placement in the Didymella/Phoma complex (Phaeosphaeriaceae, Pleosporales). Mycotaxon 93: 297-308.

Upadhyay, R, and Pavgi, MS. 1973. Morphology, development and cytology of Taphrina maculans Butler. Mycopathologia et mycologia applicata 50: 109–126.

Uzun, Y, and Kaya, A. 2018. *Rhizodiscina* Hafellner, a new genus record for Turkish Dothideomycetes. Acta Biologica Turcica 31: 141-145.

van der Nest, MA, Beirn, LA, Crouch, JA, Demers, JE, de Beer, ZW, De Vos, L, Gordon, TR, Moncalvo, J-M, Naidoo, K, Sanchez-Ramirez, S, et al. 2014. Draft genomes of *Amanita jacksonii, Ceratocystis albifundus, Fusarium circinatum, Huntiella omanensis, Leptographium procerum, Rutstroemia sydowiana*, and *Sclerotinia echinophila*. IMA Fungus 5: 472-486.

Vaughan-Martini, A, and Martini, A. 2011a. Chapter 61 - *Saccharomyces* Meyen ex Reess (1870). In The Yeasts (Fifth Edition), Kurtzman CP, Fell JW, andBoekhout T (eds), Elsevier, London: 733-746.

Vaughan-Martini, A, and Martini, A. 2011b. Chapter 66 - *Schizosaccharomyces* Lindner (1893). In The Yeasts (Fifth Edition), Kurtzman CP, Fell JW, andBoekhout T (eds), Elsevier, London: 779-784.

Voglmayr, H, Castlebury, L, and Jaklitsch, W. 2017. *Juglanconis* gen. nov. on Juglandaceae, and the new family Juglanconidaceae (Diaporthales). Persoonia: Molecular Phylogeny and Evolution of Fungi 38: 136.

Voglmayr, H, and Jaklitsch, WM. 2011. Molecular data reveal high host specificity in the phylogenetically isolated genus Massaria (Ascomycota, Massariaceae). Fungal Diversity 46: 133-170.

Watanabe, K, Nozawa, S, Hsiang, T, and Callan, B. 2018. The cup fungus *Pestalopezia brunneopruinosa* is *Pestalotiopsis gibbosa* and belongs to Sordariomycetes. PLOS ONE 13: e0197025.

Wehmeyer, LE. 1954. Perithecial development in Pleospora trichostoma. Botanical Gazette 115: 297-310.

Williamson, SM, Hodges, CS, and Sutton, TB. 2004. Re-examination of *Peltaster fructicola*, a member of the apple sooty blotch complex. Mycologia 96: 885–890.

Woudenberg, JHC, Groenewald, JZ, Binder, M, and Crous, PW. 2013. *Alternaria* redefined. Studies in Mycology 75: 171–212.

Wu, H-X, Schoch, CL, Boonmee, S, Bahkali, AH, Chomnunti, P, and Hyde, KD. 2011. A reappraisal of Microthyriaceae. Fungal Diversity 51: 189–248.
